# Coulomb interactions and migrating Dirac cones imaged by local quantum oscillations in twisted graphene

**DOI:** 10.1038/s41567-025-02786-z

**Published:** 2025-02-14

**Authors:** Matan Bocarsly, Indranil Roy, Vishal Bhardwaj, Matan Uzan, Patrick Ledwith, Gal Shavit, Nasrin Banu, Yaozhang Zhou, Yuri Myasoedov, Kenji Watanabe, Takashi Taniguchi, Yuval Oreg, Daniel E. Parker, Yuval Ronen, Eli Zeldov

**Affiliations:** 1 Department of Condensed Matter Physics, Weizmann Institute of Science, Rehovot, Israel; 2 Department of Physics, Harvard University, Cambridge, MA USA; 3 Department of Physics and Institute for Quantum Information and Matter, California Institute of Technology, Pasadena, CA USA; 4 Walter Burke Institute of Theoretical Physics, California Institute of Technology, Pasadena, CA USA; 5 Research Center for Electronic and Optical Materials, National Institute for Materials Science, Tsukuba, Japan; 6 Research Center for Materials Nanoarchitectonics, National Institute for Materials Science, Tsukuba, Japan; 7 Department of Physics, University of California at San Diego, La Jolla, CA USA; 8 Department of Physics, University of California, Berkeley, CA USA

**Keywords:** Topological matter, Condensed-matter physics, Ferromagnetism, Quantum mechanics

## Abstract

Flat-band moiré graphene systems are a quintessential platform for investigating correlated phases of matter. Various interaction-driven ground states have been proposed, but despite extensive experimental effort, there has been little direct evidence that distinguishes between various phases, in particular near the charge neutrality point. Here we probe the fine details of the density of states and the effects of Coulomb interactions in alternating-twist trilayer graphene by imaging the local thermodynamic quantum oscillations with a nanoscale scanning superconducting quantum interference device. We find that the charging self-energy due to occupied electronic states is most important in explaining the high-carrier-density physics. At half-filling of the conduction flat band, we observe ferromagnetic-driven symmetry breaking, suggesting that it is the most robust mechanism in the hierarchy of phase transitions. Near charge neutrality, where exchange energy dominates over charging self-energy, we find a nematic semimetal ground state, which is theoretically favoured over gapped states in the presence of heterostrain. In this semimetallic phase, the flat-band Dirac cones migrate towards the mini-Brillouin zone centre, spontaneously breaking the threefold rotational symmetry. Our low-field local quantum oscillation technique can be used to explore the ground states of many strongly interacting van der Waals systems.

## Abstract (web summary)

Our understanding of the phase diagram of twisted graphene structures is incomplete. Now, twisted trilayer graphene is examined using a technique that locally images quantum oscillations and shows that a nematic semimetal is favoured at low density.

## Main

**Fig. 1:**
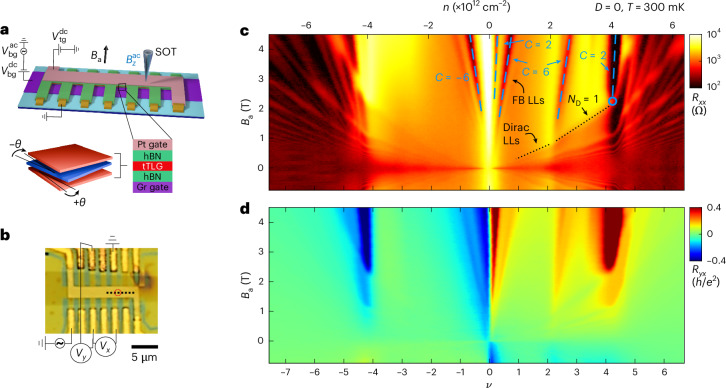
Transport measurements in tTLG. **a**, Top, schematic of the tTLG sample. Top platinum (Pt) gate and bottom graphite (Gr) gate voltages, ${V}_{\rm{tg}}^{\;\rm{dc}}$ and ${V}_{\rm{bg}}^{\;\rm{dc}}+{V}_{\rm{bg}}^{\;\rm{ac}}$, and the corresponding a.c. magnetic field ${B}_{z}^{\rm{ac}}$ imaged by the scanning SOT are indicated. Bottom, schematic of alternating tTLG. **b**, Optical image of the tTLG sample with indicated contacts where the longitudinal voltage ${V}_{{x}}$ and transverse voltage ${V}_{{y}}$ are measured. The dotted black line marks the line cuts in Figs. 2 and 3, and the red circle marks the point measurements in Fig. 4. **c**, Longitudinal resistance ${R}_{{xx}}$ versus carrier density $n$ (top axis) and magnetic field ${B}_\mathrm{a}$ at temperature *T* = 300 mK shown on a logarithmic scale. Two Landau fans emanate from the CNP: a steep fan originating from the FB LLs and a shallow fan originating from the Dirac LLs (Extended Data Fig. 1a and Supplementary Information Section I). Dashed blue lines follow the ${R}_{{xx}}$ minima labelled by their Chern number *C*, defined by the slope. The blue circle marks where the first Dirac LL, *N*_D_ = 1, crosses the top of the FB. **d**, Transverse resistance ${R}_{{yx}}$ versus filling factor $\nu$ and ${B}_\mathrm{a}$ in units of $h/{e}^{2}$.

Moiré materials offer a combination of strongly correlated phases together with high experimental tunability. They provide a controllable window into the mechanisms and origins of interacting quantum materials. The example of magic-angle twisted bilayer graphene (MATBG)^1–10^ led the way. It has flat bands (FBs) whose electronic interactions produce prominent correlated insulating states and unconventional superconductivity. Subsequently, the same basic phenomena were discovered in alternating-twist trilayer graphene (tTLG)^11–18^. In that material, the top and bottom layers are twisted with the same angle $\theta$ relative to the middle layer (Fig. 1a). This similarity is a consequence of mirror symmetry, which splits the tTLG band structure (BS) into two decoupled sectors^19,20^, MATBG-like FBs and a bystander monolayer-graphene-like Dirac cone. The extra Dirac cone sector can be used to probe the correlated physics in the FB sector^17^. Additionally, the application of a transverse displacement field *D* breaks the mirror symmetry and hybridizes the two sectors, thus providing a different degree of in situ tunability. Indeed, possibly due to the larger magic angle, the correlated insulators and superconductors in tTLG are more stable than in MATBG^21^, making it a prime platform for exploring the nature of these phases.

However, despite extensive theoretical and experimental efforts, the identity of many phases—including the phase at the charge neutrality point (CNP)—remains unknown. Theoretical studies^22–33^, including self-consistent Hartree–Fock (HF) and strong coupling analytics, have considered symmetry-broken states of magic-angle FB systems at integer fillings. Numerous ground states have been proposed, including the valley polarized state, the valley Hall state and Kramer’s intervalley coherent (KIVC) state^23^, all of which are gapped, and a gapless nematic semimetal (NSM) that spontaneously breaks the ${C}_{3}$ symmetry^24^. However, there is little direct experimental evidence distinguishing between these orders, especially at the CNP. Transport studies have revealed Chern insulators at some integer fillings^3,8,10^, but many of the underlying symmetries of the various many-body states remain unclear. More broadly, transport measurements are less well suited for identifying ground states as they do not directly probe the density of states (DOS), and merely a few techniques directly probe the BS. One example is scanning tunnelling microscopy, which has recently revealed an incommensurate Kekulé spiral order at $\nu =\pm 2$ in twisted graphene systems^18,34,35^. From a theoretical perspective, the ground state at the CNP plays a central role in determining the entire phase diagram. Yet, barring observations of $\mathrm{C}_{3}$-symmetry breaking by scanning tunnelling microscopy in some systems^18,36–39^, there has been little experimental exploration of CNP ground states. This provides strong motivation for developing experimental techniques that can probe charge neutrality.

Here we study the local thermodynamic de Haas–van Alphen quantum oscillations (QOs)^40,41^ in tTLG slightly away from the magic angle. We detected QOs in the Dirac sector down to a magnetic field of 56 mT. This was facilitated by the low DOS of the Dirac sector and the lower sensitivity of the local measurements to the effects of disorder. In addition to providing high-energy resolution, these oscillations allowed us to probe the ground-state physics and to directly visualize the symmetry breaking and BS renormalization due to the direct and exchange Coulomb interactions in the FBs.

By employing self-consistent mean-field Hartree calculation, we found that the FB dispersion was substantially renormalized by Coulomb repulsion and that the Hartree interaction term describes well the evolution of the bandwidth with filling^15,42–44^. At half-filling of the conduction FB, we found clear evidence of spontaneous flavour-symmetry breaking^6,45^, which suggests that the Stoner-polarized symmetry-broken phase is the parent state of the pervasive $\nu =2$ correlated insulator in MATBG^46,47^. Most importantly, near the CNP we found strong evidence that the CNP ground state is an exchange-interaction-driven NSM that spontaneously breaks $\mathrm{C}_{3}$ symmetry. This phase is not generally favoured theoretically^23^. However, even a small amount of heterostrain can make it energetically favourable^48^. Our findings of a semimetallic ground state provide invaluable insights into the long-standing puzzle regarding the absence of an observable gap at the CNP in MATBG.

## Transport measurements

**Fig. 2:**
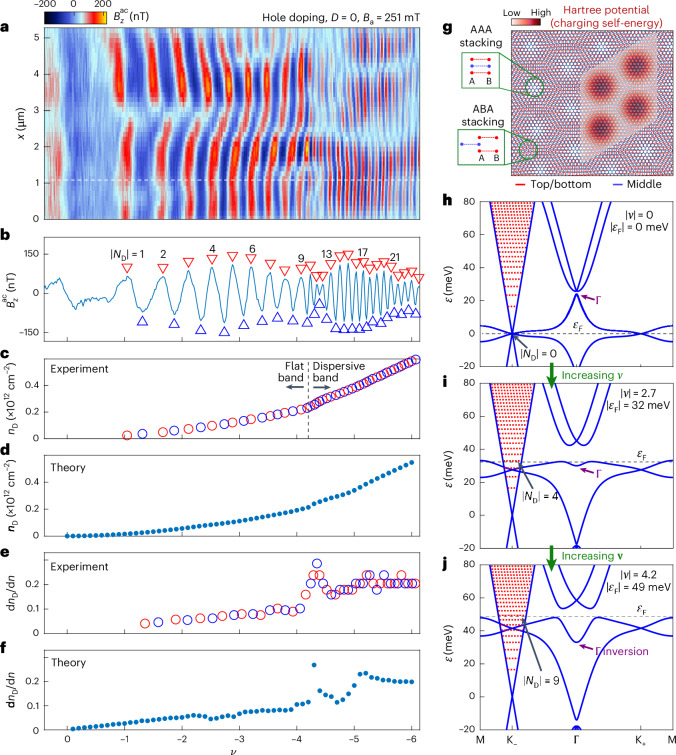
Imaging Dirac LLs and Hartree interactions. **a**, ${B}_{z}^{\rm{ac}}\left(x\right)$ versus hole carrier density measured along the dotted black line in Fig. 1c at $D=0$ and ${B}_\mathrm{a}=251$ mT, showing QOs due to LLs in the Dirac band. **b**, Cross section of ${B}_{z}^{\rm{ac}}$ versus density along the white dashed line in **a**. Red (blue) triangles mark maxima (minima) in the local magnetization due to equilibrium currents flowing in the compressible (incompressible) states in Dirac LLs, labelled by their $\left|{N}_\mathrm{D}\right|$. **c**, Carrier density in the Dirac sector ${n}_\mathrm{D}$ derived from **b**. Each oscillation in ${B}_{z}^{\rm{ac}}$ corresponds to adding ${\Delta n}_\mathrm{D}=4{B}_\mathrm{a}/{\phi }_{0}$ carriers to the Dirac sector (where $\phi_0$ is the flux quantum). Red (blue) circles correspond to ${n}_\mathrm{D}$ extracted from maxima (minima) points in **b**. **d**, ${n}_\mathrm{D}$ derived from the Hartree calculation. It is in good agreement with **c**. **e**, Numerical derivative of **c**, $\mathrm{d}{n}_\mathrm{D}/\mathrm{d}n$, which reflects the fraction of carriers added to the system that go to the Dirac sector, as a function of density. Red (blue) circles correspond to derivatives between the adjacent maxima (minima) points in **b**. **f**, $\mathrm{d}{n}_\mathrm{D}/\mathrm{d}n$ extracted from theoretical calculation in **d**. It is in good agreement with **e**. **g**, Schematic of the moiré pattern due to the twist angle $\theta$ between the middle and top/bottom layers. The AAA and ABA stacked regions are zoomed in. The wavefunctions of the FB electrons are mostly localized to the AAA regions, leading to a charging self-energy described by the periodic Hartree potential shown by the heat map and to a doping-dependent BS. **h**–**j**, BS line cuts through high symmetry points of tTLG with Hartree interaction at $\left|\nu \right|=0$ (**h**), 2.7 (**i**) and 4.2 (**j**) showing how the BS changes with doping. At $\nu =0$, the Hartree interaction term is zero and the BS is equivalent to a single-particle continuum model. As $\nu$ is increased, the Hartree interaction increases approximately linearly with filling (Supplementary Video 1). Purple arrows indicate the inversion of the $\Gamma$ point energy from the top of the conduction band at small $\nu$ to the bottom of the conduction FB at large $\nu$. The red dotted lines indicate the LL energy levels ${\varepsilon }_\mathrm{N}^\mathrm{D}$ in the Dirac sector at ${B}_\mathrm{a}=251$ mT. The black dashed line shows the evolution of the Fermi energy $\left|{\varepsilon }_\mathrm{F}\right|$ with doping and the corresponding Dirac LL $\left|{N}_\mathrm{D}\right|$ that it crosses. Source data

**Extended Data Fig. 1:**
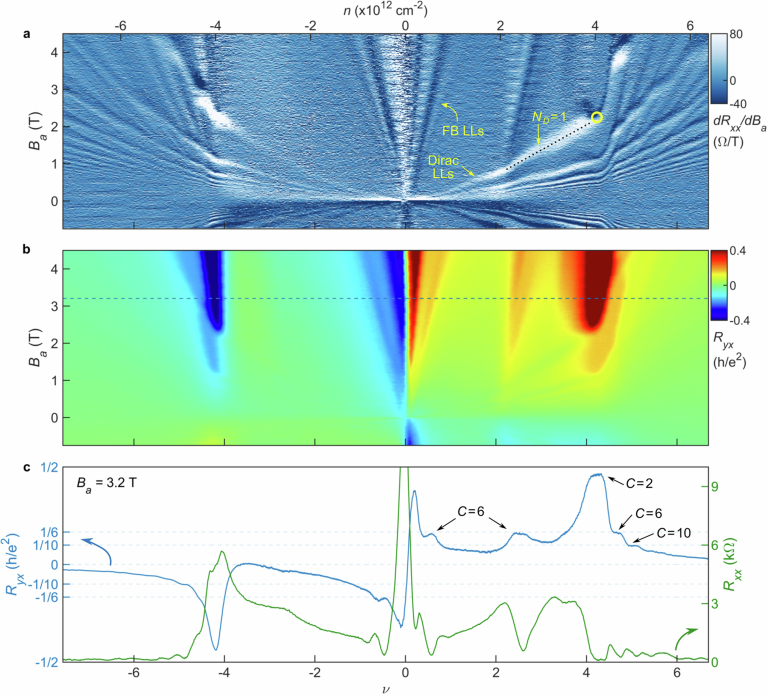
Transport and Chern numbers. **a**, Numerical derivative of ${R}_{xx}$ shown in Fig. 1c, $d{R}_{xx}/d{B}_{a}.$ Here the Dirac LLs are readily discerned. The yellow circle marks the crossing of the ${N}_{D}=1$ Dirac LL with the top of the FB. **b**,$\,{R}_{yx}$ reproduced from Fig. 1d. **c**, ${R}_{xx}$ (right axis) and ${R}_{yx}$ (left axis) vs. $\nu$ at ${B}_{a}=$ 3.2 T along the dashed lines in (b). Quantized ${R}_{yx}$ values are indicated with their Chern numbers that match the accompanying slopes of the ${R}_{xx}$ minima in Fig. 1c.

The tTLG sample was fabricated using the dry-transfer method and encapsulated in hexagonal boron nitride (hBN; Fig. 1a,b and Methods). The d.c. voltages ${V}_{\rm{tg}}^{\;\rm{dc}}$ and ${V}_{\rm{bg}}^{\;\rm{dc}}$ applied to the top and bottom gates allowed us to control the carrier density $n$ and $D$ simultaneously. Transport measurements of ${R}_{{xx}}$ and ${R}_{{yx}}$ at constant *D* = 0, were performed at a temperature *T* = 300 mK as a function of the applied out-of-plane magnetic field ${B}_\mathrm{a}$ and $n$, respectively (Fig. 1c,d). At *D* = 0, the mirror symmetry preserved the decoupling of the FB sector and the Dirac cone (Fig. 2h)^19,20^. As such, two Landau fans emanate from the CNP: a dense fan emerging at large ${B}_\mathrm{a}$ reflecting Landau levels (LLs) in the FBs and a highly dispersing fan at low ${B}_\mathrm{a}$ reflecting the LLs arising from the Dirac sector (Fig. 1c and Extended Data Fig. 1a). As the Dirac band is populated in parallel with the FBs, it shunts any gapped FB state, including the fourfold filling of the FBs (which is highly resistive in MATBG). Therefore, extracting information from transport demands a careful analysis of the two sectors (Supplementary Information Section I)^17^.

The Chern number sequence in the Dirac band is ${C}_\mathrm{D}=4{N}_\mathrm{D}+2$, and the energies of the corresponding LLs are

1 $${\varepsilon }_\mathrm{N}^\mathrm{D}=\operatorname{sgn}\left({N}_\mathrm{D}\right){v}_\mathrm{F}\sqrt{2e{\hbar }\left\vert{N}_\mathrm{D}\right\vert{B}_\mathrm{a}}.$$

Here, *e* is the elementary charge, ℏ the reduced Planck constant, ${v}_\mathrm{F}$ is the Dirac Fermi velocity and ${N}_\mathrm{D}=0,\pm 1,\pm 2,\ldots$ is the LL index in the Dirac sector. The ${N}_\mathrm{D}=0$ LL resides at the CNP with ${C}_\mathrm{D}=2$, and the half-filled compressible ${N}_\mathrm{D}=1$ LL (dotted line in Fig. 1c) crosses the edge of the FB at ${n}_{0}=4.03\times {10}^{12}$ cm^−2^ and ${B}_\mathrm{a}=2.25$ T (blue circle in Fig. 1c). At this point in phase space, the ${N}_\mathrm{D}=0$ LL is filled along with half of the ${N}_\mathrm{D}=1$ LL. Therefore, the density in the Dirac sector is ${n}_\mathrm{D}=4{B}_\mathrm{a}/{\phi }_{0}=0.22\times {10}^{12}$ cm^−2^, where ${\phi }_{0}=h/e$ is the flux quantum. The density in the FBs is then ${n}_\mathrm{F}={n}_{0}-{n}_\mathrm{D}=3.81\times {10}^{12}$ cm^−2^, which we assigned to be the filling factor $\nu =4$ in the FB. This allowed us to determine the twist angle of the tTLG device, $\theta =1.3^{\circ}$ (Supplementary Information Section I), which is slightly below the magic angle.

Inserting ${B}_\mathrm{a}=2.25$ T and ${N}_\mathrm{D}=1$ (Fig. 1c, blue circle) into equation (1), we found ${\varepsilon }_\mathrm{F}=W=49.4$ meV, where $W$ is the bandwidth of the conduction FB. This is substantially larger than $W$ calculated from the non-interacting continuum model of $W\approx24$ meV (Fig. 2h), indicating that Coulomb interactions are occurring as discussed below. Note that the hole spectrum has no pronounced features within the FB, whereas the electron spectrum has a distinct dip in ${R}_{{xx}}$ above $\nu =2$, along with non-trivial ${R}_{{yx}}$ (Fig. 1d). Additionally, the slope of the Dirac LLs changes as they cross the $\nu =2$ peak in ${R}_{{xx}}$ (black dotted lines in Fig. 1c and also Extended Data Fig. 1a), indicating strongly correlated behaviour in the FBs^11,12,17^, as elaborated below.

## Imaging QOs and the Dirac LLs

Under small ${B}_\mathrm{a}$, the dense LLs in the Dirac band serve as a powerful built-in ruler and spectrometer for probing the FB physics. We used a scanning superconducting quantum interference device fabricated on the apex of a sharp pipette (SQUID-on-tip, SOT)^49^ to detect the de Haas–van Alphen QOs associated with Dirac LLs at ${B}_\mathrm{a}\lesssim250$ mT. This approach, in addition to offering a fine energy scale, provides position-dependent spectroscopy on the nanoscale^40^. An indium SOT of about 160 nm diameter was scanned at a height of $h\approx180$ nm above the sample surface (Fig. 1a) at $T=300$ mK (Methods). In addition to the d.c. voltages applied to the gates, a small a.c. voltage ${V}_{\rm{bg}}^{\;\rm{ac}}$ of 85 mV r.m.s. was applied to the bottom gate, which modulated the carrier density by ${n}^{\rm{ac}}$ corresponding to ${\nu }^{\rm{ac}}=0.03$ r.m.s., and the resulting ${B}_{z}^{\rm{ac}}\left(x,y\right)={n}^{\rm{ac}}(\mathrm{d}{B}_{z}/\mathrm{d}n)$ was imaged across the sample.

We explored first the valence FB upon hole doping. A simpler picture was expected, as no symmetry breaking was observed in transport. We measured the evolution of ${B}_{z}^{\rm{ac}}\left(x\right)$ with carrier density at $D=0$ (Fig. 2a) by repeated scanning along the black dotted line in Fig. 1b, while incrementing $\nu$ in 0.02 steps at ${B}_{a}=251$ mT. The oscillations in ${B}_{z}^{\rm{ac}}$ reflect the de Haas–van Alphen QOs in the magnetization due to alternating diamagnetic and paramagnetic equilibrium currents in the compressible and incompressible LL states^50^. Owing to the high DOS in the FB sector and the low ${B}_\mathrm{a}$, only oscillations from Dirac LLs with larger energy gaps are discerned^41,50^. As $|\nu |$ is increased, the frequency of the ${B}_{z}^{\rm{ac}}\left(x\right)$ oscillations increases, and at $\left|\nu \right|\approx4.2$, there is an abrupt jump in the frequency, as the Fermi energy ${\varepsilon }_\mathrm{F}$ enteres the dispersive bands. The spatial variation of the oscillations reflects the inherent disorder in the sample.

**Extended Data Fig. 2:**
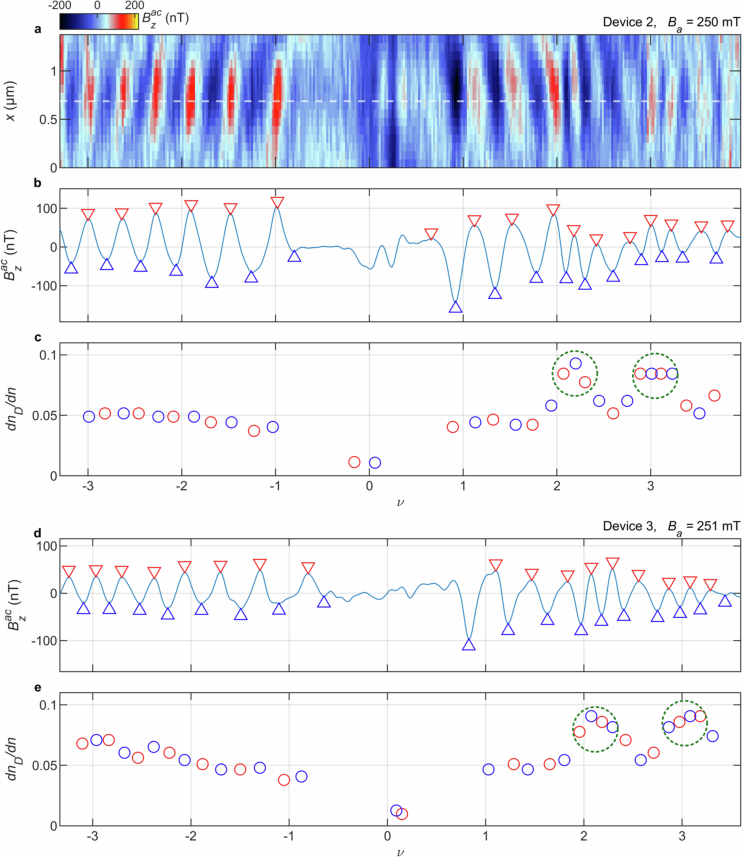
QOs in Devices 2 and 3 (*θ* ≈ 1.5°). **a**,$\,{B}_{z}^{ac}(x,\nu )$ in Device 2 at $D=0$ and ${B}_{a}=$ 250 mT upon hole and electron doping. **b**, Cross-section of ${B}_{z}^{ac}(\nu )$ along the white dashed line in (a). **c**, Fraction of carriers added to the system that go to the Dirac sector, $d{n}_{D}/dn\,$ vs. doping, extracted from (b). Green circles highlight peaks in $d{n}_{D}/dn$ near $\nu =2$ and 3 indicating symmetry breaking. **d**, ${B}_{z}^{ac}(\nu )$ in Device 3. **e**, $d{n}_{D}/dn\,$ vs. doping extracted from (d). Green circles highlight peaks in $d{n}_{D}/dn$ near $\nu =$ 2 and 3.

Figure 2b shows a cross section of ${B}_{z}^{\rm{ac}}$ as a function of doping along the dashed white line in Fig. 2a. Each maximum (red triangle) marks the centre of the ${N}_\mathrm{D}$ compressible LL whereas each minimum (blue triangle) marks the incompressible state above the same LL. In the range $\left|\nu \right|\lesssim4.2$, where the Dirac LLs and FBs coexist, nine maxima were observed, $\left|{N}_\mathrm{D}\right|=9$. Using the Dirac LLs as an energy ruler (equation (1)), we found that the FB is fully occupied when $\left|{\varepsilon }_\mathrm{F}\right|$ reaches ${\varepsilon }_{9}^\mathrm{D}=49.5$ meV. This large derived $W$ of the FB is in good agreement with transport data and is much larger than the calculated single-particle $W\approx24$ meV, emphasizing the key role of interactions. Similar behaviour can be seen in samples 2 and 3 in Extended Data Fig. 2 (Supplementary Information Section II).

Each QO period reflects the filling of another Dirac LL and an increase in the Dirac sector carrier density ${n}_\mathrm{D}$ by $\Delta {n}_\mathrm{D}=4{B}_\mathrm{a}/{\phi }_{0}=2.4\times {10}^{10}$ cm^−2^. Therefore, by counting the periods upon increasing the total carrier density $n$, we directly determined ${n}_\mathrm{D}$ as a function of doping (Fig. 2c). In the FB region ($\left|\nu \right|\lesssim4.2$), ${n}_\mathrm{D}$ grows monotonically with filling, reaching ${n}_\mathrm{D}\approx0.22\times {10}^{12}$ cm^−2^, in agreement with the value extracted from transport. Upon doping the dispersive bands ($\left|\nu \right|\gtrsim4.2$), there was a sharp increase in the slope. This is seen clearly for the derivative $\mathrm{d}{n}_\mathrm{D}/\mathrm{d}n$ in Fig. 2e, which can be understood as the fraction of electrons that populate the Dirac sector as carriers are added to the system. Such large values of ${n}_\mathrm{D}$ and $\mathrm{d}{n}_\mathrm{D}/\mathrm{d}n$ cannot be accounted for in a single-particle picture and, therefore, interactions must be considered.

## Charging self-energy and doping-dependent BS

To account for interactions, we began by considering the charging self-energy, which was modelled by a self-consistent calculation of the Hartree term (Supplementary Information Section IV.b). As the wavefunctions of the FB carriers are mostly localized to the AAA regions of the moiré pattern (Fig. 2g), an electron added to the system sees the charge build-up on the AAA sites as a background periodic potential^43,51,52^. The strength of the periodic Hartree potential ${V}_\mathrm{H}$ (equation (11) in Supplementary Information Section IV.b) is proportional to $n$, which leads to a doping-dependent BS, as shown in Fig. 2h–j (Supplementary Video 1 and Supplementary Information Sections IV and VI for BS calculations and parameters). Particle–hole symmetry allowed us to refer to the conduction band for convenience. At the CNP, the charging self-energy is zero, and the states at $\Gamma$ are at the highest energy in the conduction FB (Fig. 2h, purple arrow). Upon increasing the carrier density, the charging self-energy increases, and states away from $\Gamma$, which are mostly localized to the AAA regions, have their energies increased due to electronic build-up, eventually causing an inversion of the $\Gamma$ point to reside at the bottom of the conduction FB (Fig. 2j, purple arrow). This effect is accompanied by a large increase of the FB bandwidth relative to the non-interacting single-particle $W$ (Fig. 2h)^15,52^.

From the Hartree calculation, we extracted the evolution of ${\varepsilon }_\mathrm{F}$ with filling. It crosses the top of the flat conduction band at an energy ~49 meV consistent with $W$ extracted from the experiment. As the Dirac cone is static, ${\varepsilon }_\mathrm{F}$ directly translates into the Dirac cone density, ${n}_\mathrm{D}={4S}_\mathrm{D}/4{\uppi }^{2}=\uppi {k}_\mathrm{F}^{2}/{\uppi }^{2}=(1/\uppi){\left({\varepsilon }_\mathrm{F}/{\hslash }{v}_\mathrm{F}\right)}^{2}$, where ${S}_\mathrm{D}$ is the $k$ space area of the Dirac cone, ${k}_\mathrm{F}$ is the Fermi momentum and factor 4 comes from degeneracy. ${n}_\mathrm{D}$ versus doping is plotted in Fig. 2d. The data match the experimental results well (Fig. 2c). Furthermore, Fig. 2f shows the derivative $\mathrm{d}{n}_\mathrm{D}/\mathrm{d}n$, which is the fraction of carriers that populate the Dirac band upon further doping. $\mathrm{d}{n}_\mathrm{D}/\mathrm{d}n$ increases as a function of $n$, reaching values of 0.1 for $\left|\nu \right|\lesssim4.2$. In a non-interacting system, the BS is static and $\mathrm{d}{n}_\mathrm{D}/\mathrm{d}n$ would be given by the ratio of the Dirac DOS to the total DOS and be at most about 0.005 due to the extremely high DOS of the FBs, which causes ${\varepsilon }_\mathrm{F}$ to change slowly with doping. When accounting for the charging self-energy, however, the DOS of the FBs plays only a minor role, as the BS evolves with doping. The charging energy causes ${\varepsilon }_\mathrm{F}$ to increase with doping at a much faster rate, as the FBs are pushed up in energy, transferring more carriers into the Dirac sector (Fig. 2h–j and Supplementary Video 1). For $|\nu |\gtrsim4.2$, the jump in $\mathrm{d}{n}_\mathrm{D}/\mathrm{d}n$ is reproduced well by the theory in Fig. 2f, reflecting the drop in the total DOS at ${\varepsilon }_\mathrm{F}$ upon entering the dispersive bands. The agreement between the highly sensitive theoretical and experimental $\mathrm{d}{n}_\mathrm{D}/\mathrm{d}n$ for such an extended range in $\nu$, through the FBs and into the dispersive bands, is striking. This emphasizes the importance of the charging self-energy for providing a complete description of the mirror-symmetric twisted graphene family, which has the strongest superconductivity of all moiré systems^21^.

## Symmetry breaking at half-filling

**Fig. 3:**
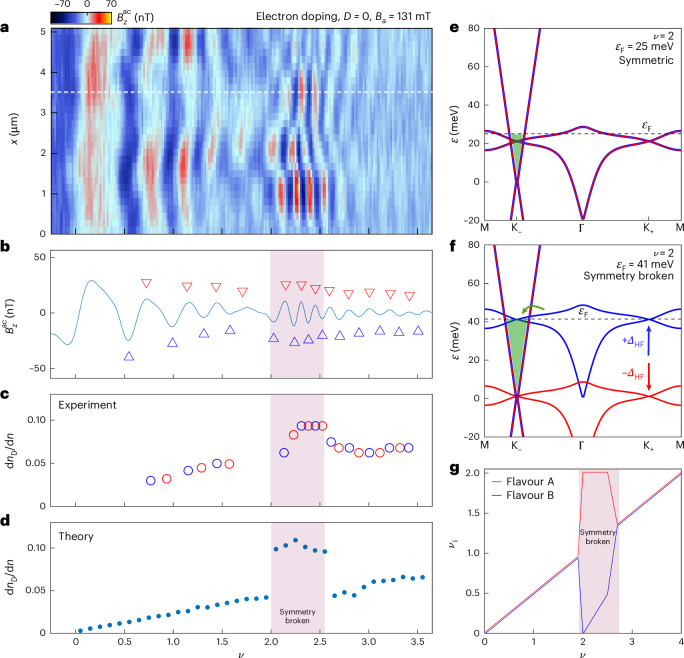
Symmetry breaking at *ν* = 2. **a**, ${B}_{z}^{\rm{ac}}\left(x,\nu \right)$ QOs as in Fig. 2 but for electron doping and at a lower ${B}_\mathrm{a}=131$ mT, which provides higher resolution for analysing the symmetry breaking at $\nu =2$. **b**, Cross section of ${B}_{z}^{\rm{ac}}\left(\nu \right)$ along the white dashed line in **a** with red (blue) triangles marking local maxima (minima). A Gaussian filter was used to smooth the data and locate the extrema. **c**, Fraction of electrons populating the Dirac sector as electrons are added to the system, $\mathrm{d}{n}_\mathrm{D}/\mathrm{d}n$, extracted from the maxima (red) and minima (blue) points in **b**. **d**, $\mathrm{d}{n}_\mathrm{D}/\mathrm{d}n$ derived from the Hartree calculation, assuming an ansatz of a $\pm 20$ meV flavour degeneracy lifting for $2\le \nu \le 2.6$ (shaded region). **e**,**f**, BS at $\nu =2$ with the proposed Stoner ansatz. Compared to the symmetric case (**e**), in the symmetry-broken state (**f**), the energy of one flavour (A) is decreased by 20 meV whereas the energy of the other flavour (B) is increased by 20 meV. In the symmetry-broken state, ${\varepsilon }_\mathrm{F}$ increases, and hence, further carriers are transferred into the Dirac cone (shaded green). **g**, Schematic of the occupation ${\nu }_{i}$, where *i* corresponds to the different flavours, with the ansatz of symmetry breaking for $2\le \nu \le 2.6$ (Supplementary Information Section III). Source data

**Extended Data Fig. 3:**
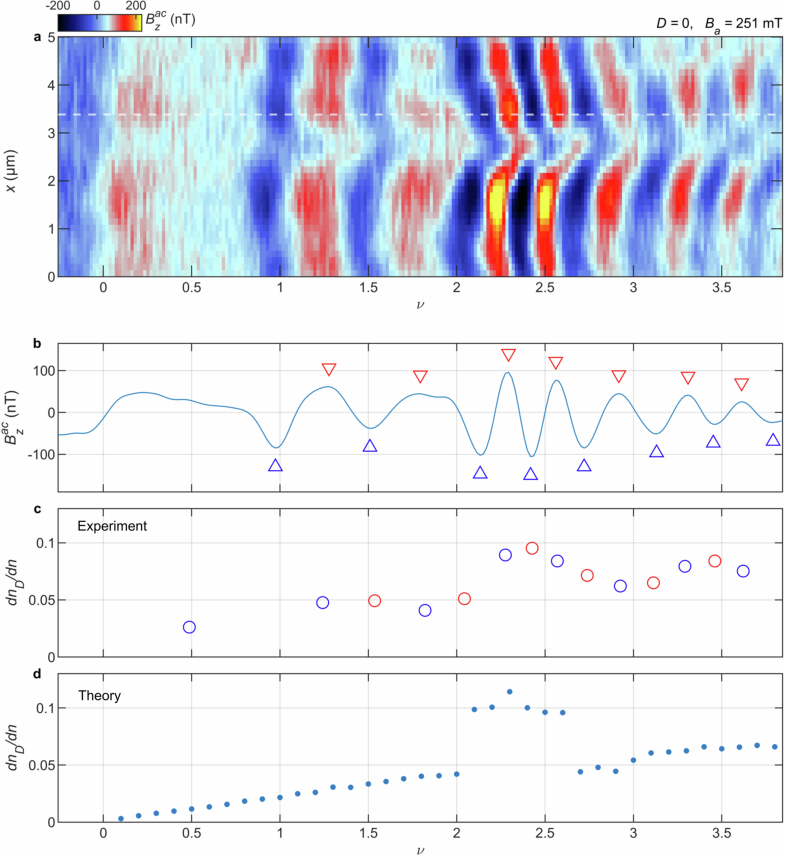
QOs in Devices 1 upon electron doping at *B*_*a*_*=* 251 mT. **a-d**, Same as in Fig. 3a-d but at a higher ${B}_{a}=$ 251 mT. **a**, ${B}_{z}^{ac}(x,\nu )$ upon electron doping. **b**, Cross-section of ${B}_{z}^{ac}(\nu )$ along the white dashed line in (a). **c**, $d{n}_{D}/dn$, extracted from (b) showing a peak above $\nu =$ 2, indicating degeneracy lifting. The resolution of the peak is poorer than in Fig. 3c due to sparser LLs at higher ${B}_{a}$. **d**, $d{n}_{D}/dn$ from the Hartree calculation.

We now explore the electron-doped spectrum, which shows signatures of symmetry breaking in transport. Figure 3a shows ${B}_{z}^{\rm{ac}}(x)$ versus $n$ at a lower ${B}_\mathrm{a}=131$ mT, which provides higher energy resolution (see Extended Data Fig. 3 for ${B}_\mathrm{a}=251$ mT data). For $\nu \lesssim2$, the ${B}_{z}^{\rm{ac}}$ oscillations are like those of their hole counterpart, whereas for $2\lesssim \nu \lesssim 2.6$, a bunching of QOs is observed along with increased intensity. This is seen more clearly in the cross section of ${B}_{z}^{\rm{ac}}$ along the dashed white line in Fig. 3a (Fig. 3b) and in the derived $\mathrm{d}{n}_\mathrm{D}/\mathrm{d}n$ (Fig. 3c). For both low ($\nu \lesssim2$) and high fillings ($\nu \gtrsim2.6$), $\mathrm{d}{n}_\mathrm{D}/\mathrm{d}n$ behaves like a valence band (Fig. 2e). However, at half-filling of the conduction FB, there is a clear jump in $\mathrm{d}{n}_\mathrm{D}/\mathrm{d}n$ (shaded region), which is absent in the valence band (Fig. 2e). This signifies that a larger fraction of carriers are populating the Dirac sector and is a clear signature of interaction-driven degeneracy lifting.

Following the data, we took as an ansatz a simple Stoner instability model, where for $2\le \nu \le 2.6$, one flavour was increased in energy by ${\varDelta}_{\rm{HF}}$ and the other was decreased by the same amount (Fig. 3e–g and Supplementary Information Section III). Based on HF calculations at even integer fillings in MATBG^20,23,24,32^, we took ${\varDelta}_{\rm{HF}}=20$ meV, which we add to the Hartree-calculated bands (Fig. 3f). The resulting occupation of the two flavours in this simple Stoner model is sketched in Fig. 3g. The shaded region marks the symmetry-broken state. Figure 3f shows that in this state, ${\varepsilon }_\mathrm{F}$ is much larger than in the symmetric state (Fig. 3e), and consequently, more carriers are transferred into the Dirac sector. This results in an increase in the calculated $\mathrm{d}{n}_\mathrm{D}/\mathrm{d}n$, as shown in Fig. 3d (shaded region), in agreement with the data in Fig. 3c. Additionally, this mechanism is consistent with the increased amplitude of the ${B}_{z}^{\rm{ac}}$ oscillations, as in the symmetry-broken state there are fewer states in the FB sector to tunnel to, resulting in lower Dirac LL broadening and a larger signal.

## Displacement field dependence

**Fig. 4:**
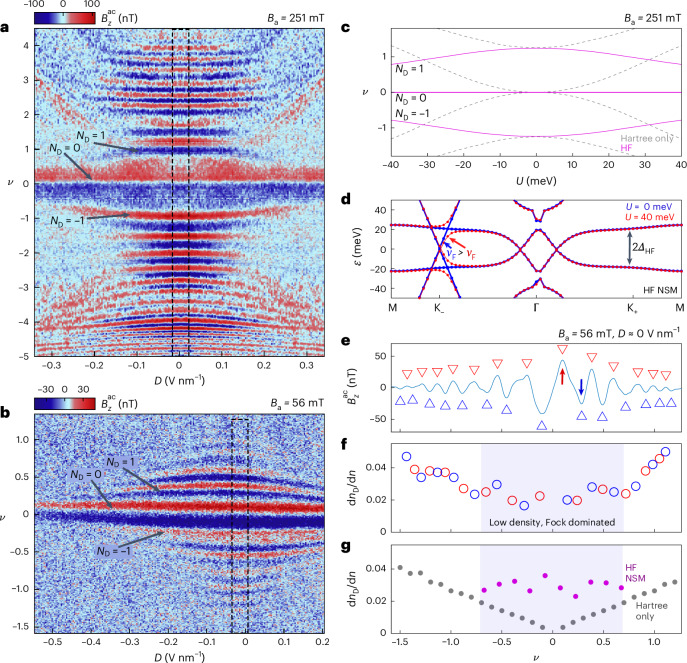
Displacement field dependence and HF calculations. **a**, ${B}_{z}^{\rm{ac}}$ QOs due to LLs in the Dirac band measured at a fixed SOT position (red circle, Fig. 1b) as a function of $\nu$ and $D$ at ${B}_\mathrm{a}=251$ mT. Diagonal streaks are an artefact coming from the bottom gate. The 0th Dirac LL has no $D$ dependence. The ${N}_\mathrm{D}=\pm 1$ LL curves towards the CNP, whereas higher LLs curve away from the CNP. **b**, Same as **a** but at ${B}_\mathrm{a}=56$ mT. The 0th LL has no $D$ dependence, but all other LLs curve towards the CNP. **c**, Simulation of Dirac LLs as a function of the layer potential difference $U$ at ${B}_\mathrm{a}=251$ mT, using the NSM state obtained from a full HF treatment (purple) and the Hartree interaction only (dashed grey). The evolution of ${N}_\mathrm{D}=0,\pm 1$ LLs in the experiment is consistent with the simulation for a NSM. **d**, BS of NSM state (Extended Data Fig. 5b) at $U=0$ (blue) and $U=40$ meV (red). FB Dirac cones migrate towards $\Gamma$. The HF gap at K_+_ and K_−_ is ${\sim}{2\varDelta}_{\rm{HF}}=40$ meV. $U$-induced hybridization between the Dirac cone and FBs (red) causes a reduced ${v}_\mathrm{F}$ in the Dirac cone below the hybridization energy (Extended Data Fig. 5a). **e**, Cross section of ${B}_{z}^{\rm{ac}}$ averaged over a range around $D\approx 0$ in the dashed box in **b**. The red arrow marks the first electron-doped paramagnetic peak, and the blue arrow marks the diamagnetic peak at ${N}_\mathrm{D}=1$. **f**, $\mathrm{d}{n}_\mathrm{D}/\mathrm{d}n$ extracted from **e**. The shaded region marks the low carrier density where the Hartree interaction is negligible and the Fock interaction dominates. **g**, Theoretical calculation of $\mathrm{d}{n}_\mathrm{D}/\mathrm{d}n$ using the Hartree interaction BS (grey) and the Fock-induced NSM BS (purple). Source data

To explore the BS evolution with displacement field $D$, we measured ${B}_{z}^{\rm{ac}}$ as a function of $\nu$ and $D$ at a fixed SOT position (red circle, Fig. 1b) at ${B}_\mathrm{a}=251$ mT (Fig. 4a) and at ${B}_\mathrm{a}=56$ mT, which has a better energy resolution with denser LLs (Fig. 4b). At ${B}_\mathrm{a}=251$ mT and $D\approx 0$ (dashed box), the QOs as a function of $\nu$ are similar to those in Figs. 2a and 3a, as expected. However, the ${B}_\mathrm{a}=56$ mT data at $D\approx 0$ (dashed box) do not scale as expected. From equation (1), it follows that the ${N}_\mathrm{D}=1$ LL at 56 mT should appear at a lower energy by a factor of the square root of the ratio of ${B}_\mathrm{a}$, that is $\sqrt{251/56}\approx 2.1$. For a high and uniform FB DOS, the ${N}_\mathrm{D}=1$ LL that appears at $\nu =0.95$ and $D\approx 0$ in Fig. 4a should appear at $\nu =0.45$ at 56 mT. Strikingly, Fig. 4b shows that the ${N}_\mathrm{D}=1$ LL appears at $\nu =0.27$, almost a factor of two below the above estimate. This strongly indicates that considering only the charging self-energy, which was used to elucidate our results thus far, cannot fully describe the correlated physics in this system close to the CNP.

**Extended Data Fig. 4:**
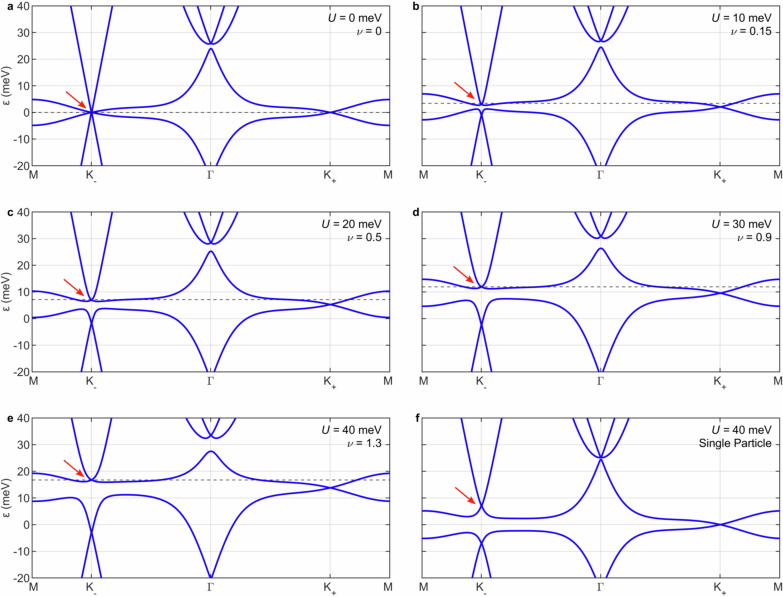
Hartree potential band structure with *U*≠0. **a-e**, Band structure line cuts for a single valley with self-consistently calculated Hartree potential for $U=0$ (**a**),$\,U=10$ (**b**), $U=20$ (**c**), $U=30$ (**d**), and $U=40$ meV (**e**). Band structure cuts are shown at the indicated values of $\nu$ for which ${\varepsilon }_{F}$ (dashed line) reaches the hybridized Dirac point where the 0^th^ LL resides, illustrating that at larger $U$ Dirac LLs appear at larger $\nu$ as shown in Fig. 4c. **f**, Band structure line cut for $U=40$ meV for a single particle calculation. Here, a similar effect is seen where the graphene and FB Dirac cones overlap and hybridize at zero energy, pushing all ${N}^{D}$ LLs up in energy.

Next, we analyse the dispersion of the Dirac LLs with $\left|D\right|$, or equivalently, as a function of the outer-layer potential difference $U$. Finite $D$ breaks the mirror symmetry and hybridizes the Dirac and FB sectors. In models where the graphene and FB Dirac cones overlap at the mini-Brillouin zone corners (K_+_ and K_−_), such as single-particle and Hartree-only systems, the hybridization occurs at the CNP (Extended Data Fig. 4). With increasing $U$, the Dirac node gets pushed to higher energy. As a result, the 0th Dirac LL along with all the higher LLs are pushed away from the CNP (Fig. 4c, grey dashed line), as occurs for ABA trilayer graphene^41^. Figure 4a indeed shows that the higher LLs disperse away from the CNP as expected. However, there are two peculiarities. First, the 0th LL stays pinned at the CNP, independent of $D$. Second, the ${N}_\mathrm{D}=\pm 1$ LLs behave contrary to expectation, curving towards the CNP.

At lower ${B}_\mathrm{a}=56$ mT (Fig. 4b), we confirm that the ${N}_\mathrm{D}=0$ LL does not disperse, and in addition, all the detectable $\left|{N}_\mathrm{D}\right| > 0$ LLs curve towards the CNP. Combining the information from Fig. 4a,b, we conclude that the Dirac LLs residing at $\left|\nu \right|\lesssim1.5$ curve towards the CNP, whereas LLs appearing at $\left|\nu \right|\gtrsim1.5$ curve away from the CNP, in sharp contrast to the Hartree prediction in Fig. 4c (grey dashed line). The behaviour of the higher carrier density is, thus, apparently captured well by considering only charging self-energy, as in Figs. 2 and 3. However, as the charging self-energy becomes small near the CNP, it is necessary to consider exchange interactions to gain insights into the underlying physics.

## Exchange interactions

**Extended Data Fig. 5:**
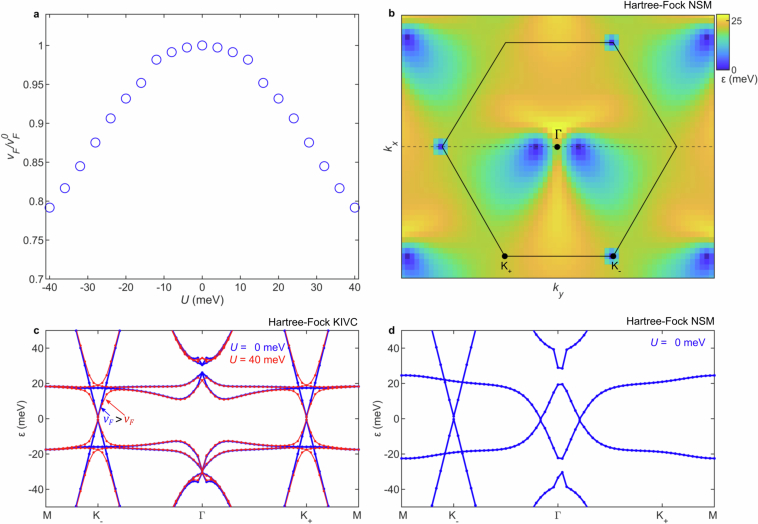
Hartree Fock calculations. **a**, Calculation of the renormalization of the Dirac cone ${v}_{F}$ at CNP as a function of $U$ in the NSM state. Values are normalized by ${v}_{F}^{0}$, ${v}_{F}$ at $U=0$, and used to plot the LL spectrum in Fig. 4c. **b**, BS of the conduction FB, $\varepsilon ({k}_{x},{k}_{y})$, of the HF NSM state at $U=0$ in an extended mBz for the $K$ valley ($K\text{'}$ is related by a 180° rotation). The system spontaneously breaks ${C}_{3}$ symmetry and FB Dirac cones migrate towards $\Gamma$, while the graphene Dirac point stay at the mBz corner. The dashed line marks the linecut along which (d) and Fig. 4d are plotted. **c**, BS of the gapped KIVC state for $U=0$ (blue) and $U=40$ meV (red). Coherence between $K$ and $K\text{'}$ folds both bands into a single mBz. Application of $U$ hybridizes the bands and renormalizes the Dirac cone ${v}_{F}$, similar to the NSM state. **d**, BS of the NSM state at $U=0$ along the back dashed line in (b). The system spontaneously breaks ${C}_{3}$ and the FB Dirac cones are shifted towards $\Gamma$.

The observed $D$ dependence reveals a lack of hybridization at the CNP, which means that graphene and FB Dirac cones do not overlap at zero energy. This can arise either due to gapping of the FB Dirac cones, namely an insulating FB state forms, or because they move away from the mini-Brillouin zone corners (K_−_ and K_+_) to maintain a semimetallic FB phase. Note that the latter automatically breaks $\mathrm{C}_{3}$ rotational symmetry, independent of the exact nature of the ground state. At the CNP, the charging self-energy is zero but strong exchange interactions in the FBs, modelled by the mean-field Fock interaction, may drive symmetry-broken orders. HF calculations at the CNP in tTLG allow many candidate symmetry-broken ground states, including the valley polarization state, the valley Hall state, KIVC and the NSM^20^. We first analyse the NSM ground state, in which exchange energy causes the FB Dirac cones to migrate towards the $\Gamma$ point (Extended Data Fig. 5b and Supplementary Information Section IV.d), spontaneously breaking $\mathrm{C}_{3}$ and no longer overlapping the graphene Dirac cones. As a result, at $U=0$, the Dirac cones and FBs intersect at an energy equal to ${\varDelta}_{\rm{HF}}\approx\pm20$ meV (Fig. 4d (blue) and Extended Data Fig. 5d). Consequently, at $U\ne 0$, the hybridization occurs at energies around ${\varDelta}_{\rm{HF}}$ (Fig. 4d, red curve), rather than near the graphene Dirac nodes as in the Hartree-only or single-particle cases (Extended Data Fig. 4). As $U$ is increased, the hybridization strength increases, effectively lowering ${v}_\mathrm{F}$ (or equivalently the slope) of the Dirac cone below the hybridization energy, as can be seen in Fig. 4d (red).

This picture successfully explains the low-energy behaviour. First, the graphene Dirac node never hybridizes, so the ${N}_\mathrm{D}=0$ LL does not disperse. Second, increasing $\left|D\right|$ decreases ${v}_\mathrm{F}$ below ${\varDelta}_{\rm{HF}}$, thereby reducing the energy of the Dirac LLs (equation (1)). This can be seen in Fig. 4c (purple), which shows the simulated Dirac LLs at ${B}_\mathrm{a}=251$ mT, by calculating the renormalized ${v}_\mathrm{F}$ as a function of $\left|D\right|$ in the NSM (Extended Data Fig. 5a). Finally, the experimentally observed boundary of $\left|\nu \right|\approx1.5$ separating the upward- and downward-dispersing Dirac LLs corresponds to a hybridization energy of about 19 meV, in good agreement with ${\varDelta}_{\rm{HF}}$ from the simulation.

Note that other HF ground states similarly result in a renormalization of ${v}_\mathrm{F}$ with $U$. These states, such as KIVC, have gapped FB Dirac cones, which also leads to hybridization at higher energies ${\varDelta}_{\rm{HF}}$ (Extended Data Fig. 5c). Therefore, the measurements of the $D$ field dependence provide irrefutable evidence that the FB Dirac cones either break $\mathrm{C}_{3}$ and migrate away from the mini-Brillouin zone corners, as in the NSM case, or have been gapped, as for KIVC.

## Ground state at the CNP

**Extended Data Fig. 6:**
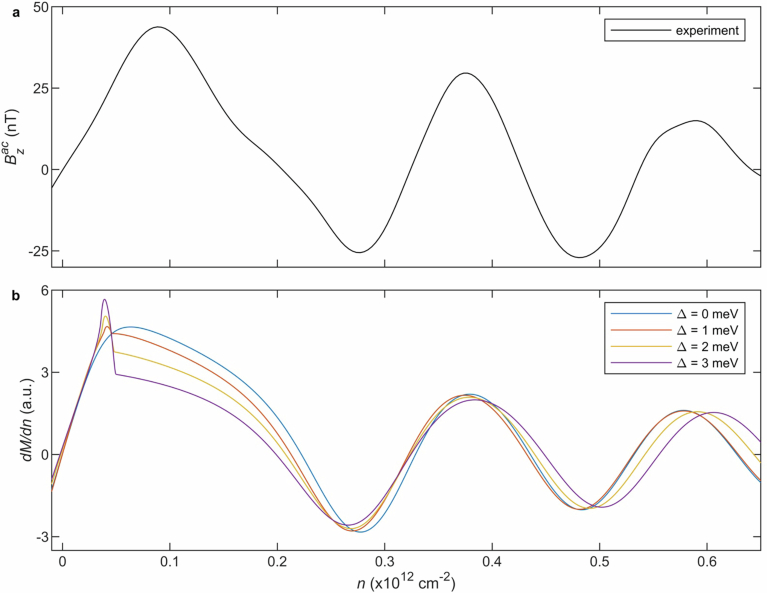
QO simulations at CNP. **a**, Zoomed-in ${B}_{z}^{ac}(n)$ near CNP at ${B}_{a}=$ 56 mT, reproduced from Fig. 4e. **b**, Simulations of QOs, $\partial M/\partial n$, arising from the Dirac band in the presence of FB described by Dirac dispersion with reduced ${v}_{F}^{FB}$ and variable FB gap $\Delta$ from 0 to 3 meV. Even a small gap of $\Delta =1$ meV is inconsistent with the shape of the measured QOs in (a).

We return focus on the $D\approx 0$ segment of Fig. 4b. Figure 4e shows a cross section of ${B}_{z}^{\rm{ac}}$ averaged around $D\approx 0$ in the dashed black box. The dense LLs at ${B}_\mathrm{a}=56$ mT carry a wealth of information about the correct ground state. Note that at this ${B}_\mathrm{a}$, the ${N}_\mathrm{D}=1$ LL resides at energy ${\varepsilon }_{1}^\mathrm{D}=7.8$ meV, observed as a diamagnetic peak in the QOs (blue arrow in Fig. 4e). Furthermore, the first paramagnetic peak, due to equilibrium currents flowing in the incompressible gap between the ${N}_\mathrm{D}=0$ and 1 LLs (red arrow in Fig. 4e), can be approximated to be at energy ${\varepsilon }_{1}^\mathrm{D}/2=3.9$ meV. Figure 4e shows that this paramagnetic peak appears at density $\nu \approx0.1$ corresponding to $n=9\times {10}^{10}$ cm^−2^, whereas only ${n}_\mathrm{D}=2{B}_\mathrm{a}/{\phi }_{0}=2.7\times {10}^{9}$ cm^−2^ carriers reside in the Dirac band. This means that at this energy, most of the carriers reside in the FB, namely the FB has a substantial DOS below 3.9 meV. Hence, if a gap exists, it must be less than a few milli-electronvolts. In fact, a more careful detailed analysis of the QO data allowed us to place an even lower bound of <1 meV on the possible gap at the CNP (Extended Data Fig. 6 and Supplementary Information Section V). This bound seems incompatible with a correlated gapped phase, where the gap is expected to be comparable to the Coulomb repulsion energy scale ~20 meV (refs. ^23,24^).

Furthermore, the separation between the upward- and downward-dispersing LLs occurs at $\left|\nu \right|\approx1.5$, which corresponds to a hybridization energy and, hence, a K-point FB gap of about ±19 meV. There is no generic theoretical reason for an order of magnitude difference between the K- and $\Gamma$-point gaps in the FBs. In the vast theoretical literature on mirror-symmetric twisted graphene, there are no ground-state candidates with a K-point gap ~19 meV and a tiny global gap. These theoretical and experimental considerations provide strong evidence for the lack of a gap in the FBs, and they effectively rule out the KIVC or any other gapped ground state at the CNP. We, thus, conclude that the CNP ground state is a $\mathrm{C}_{3}$-breaking NSM.

To verify this conclusion, we extracted $\mathrm{d}{n}_\mathrm{D}/\mathrm{d}n$ (Fig. 4f) from the QOs in Fig. 4e at ${B}_\mathrm{a}=56$ mT. For the lowest Dirac LLs, $|{N}_\mathrm{D}|\le 3$, we neglected the charging self-energy and assumed a static BS renormalized by the exchange energy. Thus, $\mathrm{d}{n}_\mathrm{D}/\mathrm{d}n$ is simply the relative DOS of the Dirac sector in the static BS, which can readily be calculated, as shown in Fig. 4g for the Hartree term only case (grey) and for the HF NSM case (purple). For large $\nu$ ($\left|{N}_\mathrm{D}\right| > 3$), the charging self-energy becomes dominant and the data follow $\mathrm{d}{n}_\mathrm{D}/\mathrm{d}n$ calculated with the Hartree term only. Close to the CNP, however, the HF NSM has the correct FB DOS needed to qualitatively match the experimental values. This provides further corroboration that the ground state at the CNP is, indeed, an NSM.

## Discussion

Generally, HF analytics find the KIVC or valley Hall state to be energetically favourable with respect to the NSM state^23^. However, even small amounts of heterostrain drive a transition that stabilizes the NSM^48^. Twisted systems in general have been shown to have substantial strain^53^, including tTLG, for which scanning tunnelling microscopy measurements show that the angle mismatch often relaxes into the mirror-symmetric configuration^14^. The apparent strain is probably related to the spatial variations in the QOs observed in Figs. 2a and 3a. Our finding of a NSM ground state at the CNP is consistent with these considerations and consistent with most experiments that find no evidence of a gap at the CNP^2,4,7,8^.

The regime of slightly off magic-angle graphene, where correlated-electron effects are weaker, provides opportunities for studying symmetry-breaking instabilities as well as their hierarchy. Our finding that the Stoner transition persists even in the absence of insulating states (Fig. 3) establishes the Stoner polarized states as the parent state for the emergence of correlated insulators. By reducing the interaction strength, we found the transition near half-filling to be most robust, in agreement with the appearance of the strongest correlated insulator state in MATBG at the same filling.

The high sensitivity of the low-magnetic-field thermodynamic QOs to the BS makes our measurement technique a powerful probe of low-energy interaction effects and fragile ground states that have yet remained unsolved in highly correlated systems. The emerging framework isolates the interaction effects at high doping, which are governed by the charging self-energy, from those at low doping where the exchange energy becomes dominant. This technique with thermodynamic QOs can readily be generalized to interacting systems that do not naturally contain a Dirac cone in the BS. One can add a Dirac band to essentially any van der Waals system by adding another monolayer graphene sheet twisted at a large angle^54,55^, such that the graphene and the system of interest share the charge density but are effectively isolated from each other at low energy.

## Methods

### Device fabrication

The hBN-encapsulated tTLG devices were fabricated using the dry-transfer method. The flakes were exfoliated onto a Si/SiO_2_ (285 nm) substrate and picked up using a polycarbonate on polydimethylsiloxane dome stamp. The number of graphene layers was determined by Raman microscopy, and the crystallographic orientations of the hBN and tTLG were determined from their straight edges. The WITec alpha300 R Raman Imaging Microscope was used to carry out Raman measurements using a wavelength of 532 nm and to cut monolayer graphene into three pieces using a 1,064 nm laser beam. During the dry-transfer process, the crystal axes of each layer of the tTLG stack were aligned by a mechanical rotation stage. After encapsulation with hBN, the stacks were released onto a pre-annealed graphite local bottom gate (~7–10 nm) patterned on an Si/SiO_2_ wafer for devices 1 and 2. For device 3, p-doped Si was used as the bottom gate. The finalized stacks were annealed in vacuum at 350 °C to release the strain. A Ti (2 nm)/Pt (12 nm) top gate was then deposited on top of the stack for device 1. For devices 2 and 3, the top gate was Ti (4 nm)/Au (14 nm). The one-dimensional contacts were etched by SF_6_ and O_2_ plasma, followed by deposition of contacts of Cr (4 nm)/Au (70 nm) in an angle-rotated e-gun evaporator. Subsequently, the Hall bar geometry was etched using SF_6_ and O_2_ plasma. Finally, the surface resist and etching residues were swept off by atomic force microscopy in contact mode.

#### Device summary

Device 1 (presented in the main text): Graphite bottom gate, Ti/Pt top gate; bottom hBN thickness ~55 nm, bottom gate capacitance ${C}_{\rm{bg}}\approx 3.4\times {10}^{11}e$ cm^−2^ V^−1^, top hBN thickness ~32 nm, top gate capacitance ${C}_{\rm{tg}}\approx 5.8\times {10}^{11}e$ cm^−2^ V^−1^ and twist angle ${\theta }_\mathrm{M}\approx 1.3^\circ$. The capacitances were calculated by fitting the slopes of Chern insulator lines in the magneto-transport measurements.

Device 2: Graphite bottom gate, Ti/Au top gate; bottom hBN thickness ~36 nm, ${C}_{\rm{bg}}\approx 5.2\times {10}^{11}e$ cm^−2^ V^−1^, top hBN thickness ~60 nm, ${C}_{\rm{tg}}\approx 3.1\times {10}^{11}e$ cm^−2^ V^−1^ and twist angle ${\theta }_\mathrm{M}\approx 1.5^\circ$.

Device 3: p-doped Si bottom gate, Ti/Au top gate; bottom hBN thickness ~36 nm, ${C}_{\rm{bg}}\approx 7.5\times {10}^{10}e$ cm^−2^ V^−1^, top hBN thickness ~60 nm, ${C}_{\rm{tg}}\approx 3.4\times {10}^{11}e$ cm^−2^ V^−1^ and twist angle ${\theta }_\mathrm{M}\approx 1.5^\circ$.

### SOT fabrication and QO measurements

The a.c. magnetic field measurements were done using an indium SOT of 160 nm effective diameter, which was fabricated as described previously^49,56,57^. The imaging was performed at $T=300$ mK using a cryogenic SQUID series array amplifier^58^. The SOT had a magnetic field sensitivity down to 10 nT Hz^−^^1/2^ and was attached to a quartz tuning fork excited at its resonance frequency of ~33 kHz for height control as described in ref. ^59^. The scanning was performed at a height of 180 nm above the sample surface.

The ${B}_{z}^{\rm{ac}}$ images were obtained with pixel size of 90 nm and acquisition time of 1 s per pixel. The measured signal ${B}_{z}^{\rm{ac}}={n}^{\rm{ac}}\left(\mathrm{d}{B}_{z}/\mathrm{d}n\right)$ is proportional to the modulation in the carrier density ${n}^{\rm{ac}}$ induced by a small a.c. voltage ${V}_{\rm{bg}}^{\;\rm{ac}}$ of 85 mV r.m.s. applied to the back gate at a frequency of $f\approx5$ kHz. To obtain the best signal-to-noise ratio from the QOs in the Dirac band, the ${V}_{\rm{bg}}^{\;\rm{ac}}$ amplitude was optimized for the lowest ${B}_\mathrm{a}=56$ mT, for which the period of the QOs $\Delta n$ was the smallest.

## Online content

Any methods, additional references, Nature Portfolio reporting summaries, source data, extended data, supplementary information, acknowledgements, peer review information; details of author contributions and competing interests; and statements of data and code availability are available at 10.1038/s41567-025-02786-z.

## Supplementary information

Supplementary Information Supplementary Discussion Sections I–VI and References.

Supplementary Video 1Doping-dependent BS. Self-consistent calculation of the mean-field Hartree interaction term, which leads to a doping-dependent BS. At $\nu =0$, the Hartree interaction term is zero and the BS is equivalent to a single-particle continuum model. As $\nu$ is increased, the Hartree term increases and the energy increases for states away from $\Gamma$. The dashed grey line marks ${\varepsilon }_\mathrm{F}$, which increases at a much faster rate. Red dashed lines correspond to the Dirac LLs at ${B}_\mathrm{a}=251$ mT, as in Fig. 2.

## Source data

Source Data Fig. 2 Statistical source data.

Source Data Fig. 3 Statistical source data.

Source Data Fig. 4 Statistical source data.

## Data Availability

The data that supports the findings of this study are available from the corresponding authors on reasonable request. Source data are provided with this paper.

## Code availability

The Hartree potential and HF calculations used in this study are available from the corresponding author on reasonable request.

## References

[1] Cao, Y. et al. Unconventional superconductivity in magic-angle graphene superlattices. *Nature* **556**, 43 (2018). 29512651 10.1038/nature26160

[2] Cao, Y. et al. Correlated insulator behaviour at half-filling in magic-angle graphene superlattices. *Nature* **556**, 80 (2018). 29512654 10.1038/nature26154

[3] Lu, X. et al. Superconductors, orbital magnets and correlated states in magic-angle bilayer graphene. *Nature* **574**, 653 (2019). 31666722 10.1038/s41586-019-1695-0

[4] Yankowitz, M. et al. Tuning superconductivity in twisted bilayer graphene. *Science* **363**, 1059 (2019). 30679385 10.1126/science.aav1910

[5] Sharpe, A. L. et al. Emergent ferromagnetism near three-quarters filling in twisted bilayer graphene. *Science* **365**, 605 (2019). 31346139 10.1126/science.aaw3780

[6] Wong, D. et al. Cascade of electronic transitions in magic-angle twisted bilayer graphene. *Nature* **582**, 198 (2020). 32528095 10.1038/s41586-020-2339-0

[7] Stepanov, P. et al. Untying the insulating and superconducting orders in magic-angle graphene. *Nature* **583**, 375 (2020). 32632215 10.1038/s41586-020-2459-6

[8] Saito, Y., Ge, J., Watanabe, K., Taniguchi, T. & Young, A. F. Independent superconductors and correlated insulators in twisted bilayer graphene. *Nat. Phys.* **16**, 926 (2020).

[9] Wu, S., Zhang, Z., Watanabe, K., Taniguchi, T. & Andrei, E. Y. Chern insulators, van Hove singularities and topological flat bands in magic-angle twisted bilayer graphene. *Nat. Mater.* **20**, 488 (2021). 33589799 10.1038/s41563-020-00911-2

[10] Grover, S. et al. Chern mosaic and Berry-curvature magnetism in magic-angle graphene. *Nat. Phys.* **18**, 885 (2022).

[11] Park, J. M., Cao, Y., Watanabe, K., Taniguchi, T. & Jarillo-Herrero, P. Tunable strongly coupled superconductivity in magic-angle twisted trilayer graphene. *Nature* **590**, 249 (2021). 33526935 10.1038/s41586-021-03192-0

[12] Hao, Z. et al. Electric field – tunable superconductivity in alternating-twist magic-angle trilayer graphene. *Science* **1138**, 1133 (2021). 10.1126/science.abg0399 33542148

[13] Christos, M., Sachdev, S. & Scheurer, M. S. Correlated insulators, semimetals, and superconductivity in twisted trilayer Graphene. *Phys. Rev. X* **12**, 21018 (2022).

[14] Turkel, S. et al. Orderly disorder in magic-angle twisted trilayer graphene. *Science* **376**, 193 (2022). 35389784 10.1126/science.abk1895

[15] Kim, H. et al. Evidence for unconventional superconductivity in twisted trilayer graphene. *Nature* **606**, 494 (2022). 35705819 10.1038/s41586-022-04715-z

[16] Zhang, Y. et al. Promotion of superconductivity in magic-angle graphene multilayers. *Science* **377**, 1538 (2022). 36173835 10.1126/science.abn8585

[17] Shen, C. et al. Dirac spectroscopy of strongly correlated phases in twisted trilayer graphene. *Nat. Mater.* **22**, 316 (2023). 36550373 10.1038/s41563-022-01428-6

[18] Kim, H. et al. Imaging inter-valley coherent order in magic-angle twisted trilayer graphene. *Nature* **623**, 942 (2023). 37968401 10.1038/s41586-023-06663-8

[19] Khalaf, E., Kruchkov, A. J., Tarnopolsky, G. & Vishwanath, A. Magic angle hierarchy in twisted graphene multilayers. *Phys. Rev. B* **100**, 85109 (2019).

[20] Ledwith, P. J. et al. TB or not TB? Contrasting properties of twisted bilayer graphene and the alternating twist *n*-layer structures (*n* = 3, 4, 5, …). Preprint at https://arxiv.org/abs/2111.11060 (2021).

[21] Park, J. M. et al. Robust superconductivity in magic-angle multilayer graphene family. *Nat. Mater.* **21**, 877 (2022). 35798945 10.1038/s41563-022-01287-1

[22] Cea, T., Walet, N. R. & Guinea, F. Electronic band structure and pinning of Fermi energy to Van Hove singularities in twisted bilayer graphene: a self-consistent approach. *Phys. Rev. B* **100**, 205113 (2019).

[23] Bultinck, N. et al. Ground state and hidden symmetry of magic-angle graphene at even integer filling. *Phys. Rev. X* **10**, 031034 (2020).

[24] Liu, S., Khalaf, E., Lee, J. Y. & Vishwanath, A. Nematic topological semimetal and insulator in magic-angle bilayer graphene at charge neutrality. *Phys. Rev. Res.* **3**, 013033 (2021).

[25] Po, H. C., Zou, L., Senthil, T. & Vishwanath, A. Faithful tight-binding models and fragile topology of magic-angle bilayer graphene. *Phys. Rev. B* **99**, 195455 (2019).

[26] Xie, M. & Macdonald, A. H. Nature of the correlated insulator states in twisted bilayer graphene. *Phys. Rev. Lett.* **124**, 97601 (2020). 10.1103/PhysRevLett.124.097601 32202880

[27] Dodaro, J. F., Kivelson, S. A., Schattner, Y., Sun, X. Q. & Wang, C. Phases of a phenomenological model of twisted bilayer graphene. *Phys. Rev. B* **98**, 075154 (2018).

[28] Phong, V. T., Pantaleón, P. A., Cea, T. & Guinea, F. Band structure and superconductivity in twisted trilayer graphene. *Phys. Rev. B* **104**, L121116 (2021).

[29] Ledwith, P. J., Khalaf, E. & Vishwanath, A. Strong coupling theory of magic-angle graphene: a pedagogical introduction. *Ann. Phys.* **435**, 168646 (2021).

[30] Po, H. C., Zou, L., Vishwanath, A. & Senthil, T. Origin of Mott insulating behavior and superconductivity in twisted bilayer graphene. *Phys. Rev. X* **8**, 031089 (2018).

[31] Hofmann, J. S., Khalaf, E., Vishwanath, A., Berg, E. & Lee, J. Y. Fermionic Monte Carlo study of a realistic model of twisted bilayer graphene. *Phys. Rev. X* **12**, 11061 (2022).

[32] Cea, T. & Guinea, F. Band structure and insulating states driven by Coulomb interaction in twisted bilayer graphene. *Phys. Rev. B* **102**, 045107 (2020).

[33] Pantaleón, P. A., Phong, V. T., Naumis, G. G. & Guinea, F. Interaction-enhanced topological Hall effects in strained twisted bilayer graphene. *Phys. Rev. B* **106**, L161101 (2022).

[34] Nuckolls, K. P. et al. Quantum textures of the many-body wavefunctions in magic-angle graphene. *Nature* **620**, 525 (2023). 37587297 10.1038/s41586-023-06226-x

[35] Kwan, Y. H. et al. Kekulé spiral order at all nonzero integer fillings in twisted bilayer graphene. *Phys. Rev. X* **11**, 41063 (2021).

[36] Choi, Y. et al. Electronic correlations in twisted bilayer graphene near the magic angle. *Nat. Phys.* **15**, 1174 (2019).

[37] Jiang, Y. et al. Charge order and broken rotational symmetry in magic-angle twisted bilayer graphene. *Nature* **573**, 91 (2019). 31365921 10.1038/s41586-019-1460-4

[38] Kerelsky, A. et al. Maximized electron interactions at the magic angle in twisted bilayer graphene. *Nature* **572**, 95 (2019). 31367030 10.1038/s41586-019-1431-9

[39] Xie, Y. et al. Spectroscopic signatures of many-body correlations in magic-angle twisted bilayer graphene. *Nature* **572**, 101 (2019). 31367031 10.1038/s41586-019-1422-x

[40] Bocarsly, M. et al. De Haas–van Alphen spectroscopy and magnetic breakdown in moiré graphene. *Science* **383**, 42 (2024). 38175887 10.1126/science.adh3499

[41] Zhou, H. et al. Imaging quantum oscillations and millitesla pseudomagnetic fields in graphene. *Nature* **624**, 275 (2023). 37993718 10.1038/s41586-023-06763-5 PMC10719110

[42] Calderón, M. J. & Bascones, E. Interactions in the 8-orbital model for twisted bilayer graphene. *Phys. Rev. B* **102**, 155149 (2020).

[43] Goodwin, Z. A. H., Vitale, V., Liang, X., Mostofi, A. A. & Lischner, J. Hartree theory calculations of quasiparticle properties in twisted bilayer graphene. *Electron. Struct.* **2**, 034001 (2020).

[44] Lewandowski, C., Nadj-Perge, S. & Chowdhury, D. Does filling-dependent band renormalization aid pairing in twisted bilayer graphene? *npj Quantum Mater.* **6**, 82 (2021).

[45] Zondiner, U. et al. Cascade of phase transitions and Dirac revivals in magic-angle graphene. *Nature* **582**, 203 (2020). 32528091 10.1038/s41586-020-2373-y

[46] Shavit, G., Berg, E., Stern, A. & Oreg, Y. Theory of correlated insulators and superconductivity in twisted bilayer graphene. *Phys. Rev. Lett.* **127**, 247703 (2021). 34951791 10.1103/PhysRevLett.127.247703

[47] Wagner, G., Kwan, Y. H., Bultinck, N., Simon, S. H. & Parameswaran, S. A. Global phase diagram of the normal state of twisted bilayer graphene. *Phys. Rev. Lett.* **128**, 156401 (2022). 35499897 10.1103/PhysRevLett.128.156401

[48] Parker, D. E., Soejima, T., Hauschild, J., Zaletel, M. P. & Bultinck, N. Strain-induced quantum phase transitions in magic-angle graphene. *Phys. Rev. Lett.* **127**, 27601 (2021). 10.1103/PhysRevLett.127.027601 34296891

[49] Vasyukov, D. et al. A scanning superconducting quantum interference device with single electron spin sensitivity. *Nat. Nanotechnol.* **8**, 639 (2013). 23995454 10.1038/nnano.2013.169

[50] Uri, A. et al. Nanoscale imaging of equilibrium quantum Hall edge currents and of the magnetic monopole response in graphene. *Nat. Phys.* **16**, 164 (2020).

[51] Rademaker, L., Abanin, D. A. & Mellado, P. Charge smoothening and band flattening due to Hartree corrections in twisted bilayer graphene. *Phys. Rev. B* **100**, 205114 (2019).

[52] Choi, Y. et al. Interaction-driven band flattening and correlated phases in twisted bilayer graphene. *Nat. Phys.* **17**, 1375 (2021).

[53] Uri, A. et al. Mapping the twist-angle disorder and Landau levels in magic-angle graphene. *Nature* **581**, 47 (2020). 32376964 10.1038/s41586-020-2255-3

[54] Hoke, J. C. et al. Uncovering the spin ordering in magic-angle graphene via edge state equilibration. *Nat. Commun.* **15**, 4321 (2024). 38773076 10.1038/s41467-024-48385-z PMC11109299

[55] Jiang, J. et al. Featuring nuanced electronic band structure in gapped multilayer graphene. Preprint at https://arxiv.org/abs/2405.12885 (2024).

[56] Anahory, Y. et al. SQUID-on-tip with single-electron spin sensitivity for high-field and ultra-low temperature nanomagnetic imaging. *Nanoscale* **12**, 3174 (2020). 31967152 10.1039/c9nr08578e

[57] Finkler, A. et al. Self-aligned nanoscale SQUID on a tip. *Nano Lett.* **10**, 1046 (2010). 20131810 10.1021/nl100009r

[58] Huber, M. E. et al. DC SQUID series array amplifiers with 120 MHz bandwidth. *IEEE Trans. Appl. Supercond.* **11**, 1251 (2001).

[59] Halbertal, D. et al. Nanoscale thermal imaging of dissipation in quantum systems. *Nature* **539**, 407 (2016). 27786173 10.1038/nature19843

