# Supplementary material for: Coulomb interactions and migrating Dirac cones imaged by local quantum oscillations in twisted graphene
Source: Nat Phys. 2025 Feb 14;21(3):421–9. doi: 10.1038/s41567-025-02786-z (PMC11908974; doi:10.1038/s41567-025-02786-z)
Supplement: Supplementary file 1 — Supplementary Discussion Sections I–VI and References. [file 41567_2025_2786_MOESM1_ESM.pdf]

nature physics

Supplementary information

https://doi.org/10.1038/s41567-025-02786-z

# Coulomb interactions and migrating Dirac cones imaged by local quantum oscillations in twisted graphene

In the format provided by the authors and unedited

## Supplementary Information

### I. Transport measurements

Four-probe transport measurements were performed at $T = 300$ mK using standard lock-in technique with an *ac* bias current of $I^{ac} = 10$ nA rms at $\approx 11$ Hz. Longitudinal and transverse resistivity ($R_{xx}$ and $R_{yx}$) are measured vs $n$ and $B_a$. From $R_{xx}$ measurements we distinguish the Dirac sector from the FBs and find the full filling of the FBs at $n_F = n_0 - n_D = 3.81 \times 10^{12}$ cm$^{-2}$. We assign this density $n_F = 4\nu = 4/A_m$, where $A_m$ is the moiré unit cell area and is connected to the twist angle $\theta$ by $A_m = \frac{\sqrt{3}a^2}{2\theta^2}$, where $a = 0.246$ nm is the graphene lattice constant. We determine the twist angle of the tTLG Device 1 of $\theta = \sqrt{\frac{\sqrt{3}a^2}{2A_m}} \approx 1.3°$, slightly below the magic angle.

The transport measurements in Fig. 1 and $B_z^{ac}$ line scan data in Figs. 2 and 3 were done at $D = 0$, while the point scan data in Fig. 4 of $B_z^{ac}$ are as a function of $\nu$ and $D$. The displacement field is defined in terms of the top gate voltage, $V_{tg}^{dc}$, and bottom gate voltage, $V_{bg}^{dc}$, as $D = \left(C_{tg}\left(V_{tg}^{dc} - V_{tg}^{0}\right) - C_{bg}\left(V_{bg}^{dc} - V_{bg}^{0}\right)\right) / 2\epsilon_0$. Here $\epsilon_0$ is the permittivity of free space, and $V_{tg}^{0}$ and $V_{bg}^{0}$ correspond to the charge neutrality of the top and bottom gates respectively obtained from transport measurements at zero magnetic field.

As the Dirac and FB sectors are populated in parallel, the total Chern number is $C = C_D + C_F$, the sum of the Dirac and FB Chern numbers. The $R_{xx} = 0$ line that emanates from the blue circle in Fig. 1c and extends to higher $B_a$, has a slope of $C = 2$. At these high $B_a$, only the $N_D = 0$ Dirac LL is occupied with $C_D = 2$, and thus at $\nu = 4$ we find that $C_F = 0$ (as expected with time reversal symmetry). This is seen further in Extended Data Fig. 1c, which shows line cuts of $R_{xx}$ and $R_{yx}$ at $B_a = 3.2$ T. Above the full filling of the FB ($\nu \gtrsim 4.2$) a sequence of quantized $R_{yx}$ and dips in $R_{xx}$ is observed with $C = 2$, 6, 10, corresponding to $C_F = 0$ and $C_D = 2$, 6, 10 (Fig. 1c). Additionally, the most prominent LL emanating from CNP is $C = 6$, unlike $C = 4$ in MATBG [1], corresponding to the sum of $C_D = 2$ and $C_F = 4$, further confirming the presence of the 0$^{\text{th}}$ LL in the Dirac cone.

Extended Data Fig. 1a shows $dR_{xx}/dB_a$ of the $R_{xx}$ data shown in Fig. 1c. The derivative data shows clearly the LLs emanating from the Dirac sector. Similar to Ref. [2] a peak in $dR_{xx}/dB_a$ appears when a compressible Dirac LL passes through a compressible FB state, whereas a dipole-like feature is observed when the FB is gapped (near $\nu = 4$), as well as in the remote bands. The yellow circle marks where the $N_D = 1$ LL crosses the top of the FB, which is used to extract the bandwidth of the FB. A clear kink in the Dirac LLs is seen upon passing through the $\nu = 2$ symmetry broken state.

A prominent electron-hole asymmetry is evident, with no correlated states observed in the valance FB. On the electron side however, at elevated fields $B_a \gtrsim 1.5$ T, a $R_{xx}$ minimum follows a $C = 6$ slope and the corresponding $R_{yx}$ is quantized with the same Chern number (Extended Data Fig. 1c). At these fields, only the $N_D = 0$ LL is filled, giving $C_D = 2$ and $C_F = 4$. A topological band with such a high Chern number $C_F$ is surprising, and might be explained by translation symmetry breaking that doubles the number of low energy bands [3], but is not the focus of this work. Our local QO measurements explore low fields, where $R_{yx}$ quantization disappears and the slope in $R_{xx}$ more closely follows a $C = 2$ line (Extended Data Fig. 1a at $B_a < 1$ T), suggesting a different ground state.

### II. Magnetization oscillations in Device 2 and 3

Similar to Device 1, thermodynamic QOs are observed in Devices 2 and 3 (Extended Data Fig. 2). Upon hole doping, a monotonic increase in $dn_D/dn$ is observed (Extended Data Figs. 2b,e), similar to Device 1 (Fig. 2). In contrast, electron doping displays jumps in $dn_D/dn$ indicating symmetry breaking. Interestingly, in

Devices 2 and 3, $dn_D\,/dn$ peaks occur both at $\nu = 2$ and 3 (green circles). This matches transport data for Device 3, where at high $B_a$ insulating states appear near $\nu = 1$, 2, and 3. This is likely because the twist angle in these devices ($\theta \approx 1.5°$) is closer to the magic angle.

## III. Observation of symmetry breaking in Device 1

Figure 3 shows a peak in $dn_D\,/dn$ signifying a symmetry breaking at $\nu = 2$ measured at $B_a = 131$ mT. The same behavior is observed at higher $B_a = 251$ mT (Extended Data Fig. 3), albeit with lower resolution due to sparser LLs. The precise symmetry broken ground state is unknow from theory, depending on the symmetries imposed. Furthermore, to understand our experimental data ($dn_D\,/dn$) we are mainly interested in the evolution of $\varepsilon_F$ which is not affected significantly by the exact form of the symmetry broken state at $\nu = 2$, but rather by the energy shift. To this end, we keep our analysis general and use as an ansatz a simple Stoner instability model where for $2 \le \nu \le 2.6$ the FB of one flavor type is increased in energy by $\Delta_{HF}$= 20 meV and the other is reduced by $\Delta_{HF}$ (Figs. 3f,g). Flavor here can include the Chern basis, intervalley coherent, or involve the graphene sublattice. No matter the actual state, the broad features will be similar and the important energy shift $\Delta_{HF}$ will appear.

## IV. Theoretical modeling of band renormalization in tTLG

### a. Continuum model

We calculate the non-interacting BS of tTLG by expanding the Bistritzer-MacDonald (BM) model [4,5], The Hamiltonian of the three decoupled layers expanded around the $K$ valley is

$$H_d \;=\; \sum_{l=1,2,3} \sum_{\boldsymbol{k}} \psi^{\dagger}_{l,\boldsymbol{k}}\, h_{l,\boldsymbol{k}}\, \psi_{l,\boldsymbol{k}}, \tag{2}$$

where $\psi_{l,\boldsymbol{k}}$ is a spinor in sublattice space of electronic annihilation operators on layer $l$ at momentum $\boldsymbol{k}$ (relative to $K$), the intralayer Hamiltonian is $h_{l,\boldsymbol{k}} = v_F e^{-\frac{1}{2}i\theta_l\sigma_z}\left(k_x\sigma_x + k_y\sigma_y\right)e^{\frac{1}{2}i\theta_l\sigma_z} + (\frac{l}{2} - 1)U$, $\theta_l$ is the twist angle applied to $l$, $U$ is the potential difference between the outermost layers, $v_F$ is the monolayer graphene Fermi velocity, and the $\sigma_i$ Pauli operators act on sublattice. In the alternating-angle tTLG case, $\theta_1 = \theta_3 = \frac{\theta}{2}, \theta_2 = -\frac{\theta}{2}$.

The interlayer tunneling is described by (neglecting tunneling between the outermost layers)

$$H_t \;=\; \sum_{j} \sum_{\boldsymbol{k}} (\psi^{\dagger}_{1,\boldsymbol{k}} + \psi^{\dagger}_{3,\boldsymbol{k}})\, T_j \psi_{2,\boldsymbol{k}+q_j} + h.c., \tag{3}$$

where the tunneling matrices are given by

$$T_j = \begin{pmatrix} w_0 & w_1 e^{-\frac{2\pi}{3}i\,(j-1)} \\ w_1 e^{\frac{2\pi}{3}i\,(j-1)} & w_0 \end{pmatrix} \tag{4}$$

and $\boldsymbol{q}_1 = L\hat{y}, \boldsymbol{q}_2 = \frac{L}{2}\left(-\sqrt{3}\hat{x} - \hat{y}\right), \boldsymbol{q}_3 = \frac{L}{2}\left(\sqrt{3}\hat{x} - \hat{y}\right), L = \frac{8\pi}{3a}\sin\frac{\theta}{2}$. Here $w_0$ and $w_1$ are the tunneling strengths in the AAA and ABA stacked regions, respectively. Due to lattice relaxation, the AAA regions shrink, and thus $w_0$ is phenomenologically lowered in comparison to $w_1$.

First, we focus on a system absent of displacement field, where mirror symmetry exists and the intralayer Hamiltonians for layers $l$ = 1 and 3 are identical. We then re-define creation and annihilation operators in a new basis, $\psi_{\pm,\boldsymbol{k}} \equiv \frac{1}{\sqrt{2}}(\psi_{1,\boldsymbol{k}} \pm \psi_{3,\boldsymbol{k}})$. The $H_d$ part of the Hamiltonian remains decoupled in this new basis, and the interlayer part $H_t$ only couples $\psi_{+,\boldsymbol{k}}$ to $\psi_{2,\boldsymbol{k}}$, with an extra factor of $\sqrt{2}$ entering the tunneling

matrix. The Hamiltonian thus is decomposed into two decoupled sectors: a Dirac-cone, and a FB sector with a modified magic angle $\theta_{tTLG} = \sqrt{2}\theta_{TBG}$ ([4]).

Shifting the momentum origin of the middle $l = 2$ layer by $\boldsymbol{q}_1$, we define the moiré lattice vectors $\boldsymbol{b}_1 = \boldsymbol{q}_3 - \boldsymbol{q}_1$, and $\boldsymbol{b}_2 = \boldsymbol{q}_3 - \boldsymbol{q}_2$. We write the total non-interacting Hamiltonian $H_0 = H_d + H_t$ as

$$H_0 = \sum_{l=1,2,3} \sum_{\boldsymbol{G}} \sum_{\boldsymbol{k}} \psi^{\dagger}_{l,\boldsymbol{k}+\boldsymbol{G}} h_{l,\boldsymbol{k}+\boldsymbol{G}} \psi_{l,\boldsymbol{k}+G} + \sum_{j} \sum_{\boldsymbol{G},\boldsymbol{G}'} \sum_{\boldsymbol{k}} f_{j;\boldsymbol{G},\boldsymbol{G}'} (\psi^{\dagger}_{1,\boldsymbol{k}+\boldsymbol{G}} + \psi^{\dagger}_{3,\boldsymbol{k}+\boldsymbol{G}}) T_j \psi_{2,\boldsymbol{k}+q_j+\boldsymbol{G}'}, \tag{5}$$

where $\boldsymbol{G}, \boldsymbol{G}'$ are reciprocal lattice vectors and $f_{j;\boldsymbol{G},\boldsymbol{G}'} = 0{,}1$ defines the nearest-neighbor-only connectivity between Dirac cones in momentum space. We find that keeping only reciprocal lattice vectors at size $6|\boldsymbol{G}|$ is sufficient.

### b. Interactions and Hartree potential

To recover interaction induced BS modifications we now take the Coulomb repulsion between electrons into account. This is done, by considering:

$$H_I = \frac{1}{2} \int d\boldsymbol{r} d\boldsymbol{r}' \bar{\rho}(\boldsymbol{r}) V(\boldsymbol{r} - \boldsymbol{r}') \bar{\rho}(\boldsymbol{r}'), \tag{6}$$

where $\bar{\rho}(\boldsymbol{r})$ is the density operator relative to charge neutrality, and the Coulomb potential is $V(\boldsymbol{r}) = e^2/4\pi\epsilon|\boldsymbol{r}|$ ($\epsilon$ is the effective dielectric constant). A Hartree approximation in sufficient to reproduce the experimentally observed density dependent shift in energy between the Dirac band and the FBs. We determine the BS by self-consistent calculation of the mean field Hamiltonian,

$$H_{MF} = H_0 + H_{Hartree}(\nu), \tag{7}$$

where the filling-dependent Hartree term is given by

$$H_{Hartree} = \frac{1}{\Omega} \sum_{\boldsymbol{k},\boldsymbol{k}',\boldsymbol{q}} V_q \langle \psi^{\dagger}_{l',\boldsymbol{k}'-\boldsymbol{q}} \psi_{l',\boldsymbol{k}'} \rangle_\nu \; \psi^{\dagger}_{l,\boldsymbol{k}+\boldsymbol{q}} \psi_{l,\boldsymbol{k}}. \tag{8}$$

Here a summation over repeated layer indices is assumed, $V_q$ is the Fourier transform of $V(\boldsymbol{r})$, and $\Omega$ is the system volume. The expectation value $\langle\, . \,\rangle_\nu$ is evaluated by finding the ground state of $H_{MF}$, $|\Psi_\nu\rangle$, and calculating $\langle\, . \,\rangle_\nu = \langle \Psi_\nu | \,.\, | \Psi_\nu \rangle - \langle \Psi_0 | \,.\, | \Psi_0 \rangle$, i.e., relative to charge neutrality.

The $\boldsymbol{q} = 0$ part of this expectation value is exactly offset by the contribution of the background charge, and is thus discarded. Due to the moiré pattern which breaks the translation symmetry on the graphene scale, momentum is only conserved up to reciprocal lattice vector $\boldsymbol{G}$. We write

$$H_{Hartree} = \frac{1}{\Omega} \sum_{\boldsymbol{G}}{}' V_G \sum_{\boldsymbol{k}'} \langle \psi^{\dagger}_{l',\boldsymbol{k}'-\boldsymbol{G}} \psi_{l',\boldsymbol{k}'} \rangle_\nu \sum_{\boldsymbol{k}} \psi^{\dagger}_{l,\boldsymbol{k}+\boldsymbol{G}} \psi_{l,\boldsymbol{k}}, \tag{9}$$

where the summation $\sum'_G$ is over the six reciprocal lattice vectors in the first star (following [6]). The value of the Coulomb repulsion is $V_G = e^2/(\epsilon a/2 \, sin\left(\frac{\theta}{2}\right))$. Further noticing the fact that $V_G \sum_{\boldsymbol{k}'} \langle \psi^{\dagger}_{l',\boldsymbol{k}'-\boldsymbol{G}} \psi_{l',\boldsymbol{k}'} \rangle_\nu$ should all be equal within this first star, we define

$$V_H \equiv \frac{1}{6\Omega} \sum{}'_G V_G \sum_{\boldsymbol{k}'} \langle \psi^{\dagger}_{l',\boldsymbol{k}'-\boldsymbol{G}} \psi_{l',\boldsymbol{k}'} \rangle_\nu, \tag{10}$$

and finally write Eq. (9) in the concise form:

$$H_{Hartree} = V_H \sum_{\boldsymbol{G}}' \sum_{\boldsymbol{k'}} \psi^\dagger_{l,\boldsymbol{k}+\boldsymbol{G}} \psi_{l,\boldsymbol{k}}. \tag{11}$$

The form of Eq. (11) suggests a simple interpretation of the Hartree band renormalization term: it is a periodic potential (with the moiré pattern periodicity) acting on the electrons by the spatially non-uniform distribution of the same electrons.

To find the value of the $\nu$-dependent Hartree potential we employ an iterative self-consistent method. An initial value $V_H^{(0)}$ is chosen. The mean-field Hamiltonian $H_{MF}$ [Eq. (7)] is then diagonalized using this initial value. We then use the ground state of the mean-field Hamiltonian to calculate the next value $V_H^{(1)}$ as in the expression above Eq. (10). This step depends explicitly on $\nu$ at which we calculate $V_H$. We repeat these steps until $V_H$ converges, typically $\sim 10$ steps.

### c. Effect of displacement field

A displacement field induces an interlayer potential, $U \neq 0$, between $l = 1$ and 3. This couples the $\psi_\pm$ sectors through a term $\sum_{\boldsymbol{k}} \psi_{+,\boldsymbol{k}} \left(-\frac{U}{2}\right) \psi_{-,\boldsymbol{k}} + h.c.$, which in turn couples the FB sector with the Dirac cone sector. As a result, the FBs and the Dirac cone hybridize at the Dirac point. $V_{Hartree}$ is calculated similarly in a self-consistent fashion with $U \neq 0$. Extended Data Figs. 4a-e show the renormalized Hartree potential BS for several values of $U$. As $U$ is increased the graphene and FB Dirac cones hybridize and are pushed to higher energy. Subsequently, $\nu$ must be increased to larger values before $\varepsilon_F$ reaches the Dirac node (red arrows), where the approximate $N_D = 0$ LL appears. This causes the general trend of Dirac LLs dispersing to higher $\nu$ as a function of $U$. The same effect occurs in the single particle picture (Extended Data Fig. 4f), and more generally in any model where the graphene and FB Dirac nodes overlap in $k$-space, namely $C_3$ is not broken or FB Dirac cones are not gapped, as discussed in the following section.

To calculate the Dirac LL spectrum at $B_a = 251$ mT, as seen in Fig. 4c (grey dashed), we track the density of carriers in the Dirac cone, $n_D$, as a function of $\nu$. At values of $\nu$ for which $n_D = 4N_D B_a/\phi_0$, the compressible $N_D$ LL is half filled corresponding to the peaks in QOs. This procedure is done for a number of values of $U$, and the results interpolated and smoothed to get the continuous spectrum shown in Fig. 4c (grey dashed).

As discussed in the main text, this Hartree only calculation is sufficient to describe the experimental observations for $|\nu| \gtrsim 1.5$, but to explain the low energy physics near CNP, inclusion of the Fock term is necessary.

### d. Full Hartree Fock calculations

In this section we describe our self-consistent Hartree Fock calculations of the low energy tTLG bands [7–10]. We use a dual-gate screened Coulomb interaction with screening length $d_s = 20$ nm and effective dielectric constant $\epsilon \approx 16 - 17$ that accounts for both hBN screening and screening from remote tTLG electrons [11], the latter modeled through static Dirac cone screening. We further use the infinite temperature subtraction scheme to address double counting of interaction effects in band-structure parameters [12,13]. The specific BS parameters used are described in the following section, although we have checked that our qualitative conclusions are robust to varying these parameters within a physically reasonable range.

The inclusion of the Fock term is crucial for symmetry breaking states. While for symmetric states the Fock dispersion only acts to renormalize the non-interacting band structure, for symmetry breaking states the dispersion is drastically altered. Previously, for $\nu = 2$, we approximated Hartree Fock dispersion of Stoner states by splitting the Hartree bands symmetrically with a phenomenological $k$-independent Fock energy $\Delta_{HF}$. This is a reasonable approximation at the Fermi momenta, which are not too close to the $\Gamma$ point at

$\nu = 2$, such that the $k$-dependence of the Fock dispersion is weak. In contrast, near CNP, the large scale and strong $k$-dependence of the Fock Hamiltonian makes it the dominant term; the Hartree term vanishes at CNP, and the Fock term dwarfs the non-interacting BS. Furthermore, the low energy dispersion of the Fock term is near $\Gamma$ for all symmetry breaking states.

While one could, with significant effort, perform self-consistent Hartree Fock across all filling factors, it is a more controlled approximation at integer filling due to the proximity to the strong-coupling limit, and it does not include, for example, the dynamical screening of flat-band electrons that is likely necessary for a quantitatively correct treatment of the metallic states. We will therefore focus our efforts at CNP, where the Fock term is most needed and most reliable.

Our goal is to investigate the influence of various CMP symmetry breaking states on the graphene Dirac cone and its LLs through self-consistent Hartree Fock. The results we find are consistent with prior works [11,14,15]. For simplicity we assume spinless electrons and we take $N = 4$ bands per valley which is the minimal number of bands needed to capture the FBs and the low-energy part of the decoupled graphene Dirac cone.

By imposing or not imposing each of $U(1)_V$ (valley conservation) and $C_2T$ (two-dimensional inversion combined with time-reversal) symmetries, we can disallow or allow different symmetry breaking states. If no symmetries are imposed, we obtain the KIVC state [8] at not too large displacement field $D$, as expected from strong coupling arguments [8,11,16–18]. The KIVC state, breaks valley conservation causing a folding of both valleys into one mBz, preserves $C_2T$, and gaps the FB Dirac cones through intervalley hybridization (Extended Data Fig. 5c). If we impose $U(1)_V$, ruling out KIVC, we either obtain a $C_3$ breaking NSM [9], or a VH state which breaks $C_2T$. The VH state gaps the graphene Dirac cone with a mass $\propto U^2$. This is inconsistent with experiment, as the graphene Dirac cone 0$^{\text{th}}$ LL does not split and disperse away from CNP with $D$ field (Fig. 4a,b). We will therefore not consider the VH state further.

Nominally the NSM is disfavored relative to KIVC as well, but becomes the ground state with small amounts of strain [9,19]. Because $C_2T$ and $U(1)_V$ is preserved, and the two FB Dirac cones have the same chirality, the Dirac cones annihilate. The $C_3$ breaking of the NSM state moves the FB Dirac cones to a close vicinity of the $\Gamma$ point (Extended Data Fig. 5b). At the $K$-point, the graphene Dirac cone is protected by a FB $K$-gap and $C_2T$ symmetry, similar to KIVC state. With $D$, the graphene Dirac cone shifts slightly, and its velocity becomes slightly anisotropic, since it perturbatively feels the $C_3$ breaking of the moiré electrons. However, it remains at zero energy [11,14,15] and hybridization occurs at $\Delta_{HF}$ in sharp contrast to the CNP hybridization in the $C_3$ symmetric case. With increasing $U$, the hybridization increases and the graphene Dirac cone $v_F$ is effectively renormalized (Fig. 4d). A similar effect happens in the KIVC state (Extended Data Fig. 5c).

To quantify this effect and facilitate qualitative comparison to experiment, we evaluate the NSM BS at various values of $U$. $v_F$ is computed with respect to $U = 0$ (Extended Data Fig. 5a), and then the $N_D = \pm 1$ LL is scaled as a function of $U$ by the renormalized $v_F$ (Fig. 4c, magenta).

Note that in the NSM the migration of the FB Dirac cones to $\Gamma$ is due to the concentration of the FB Berry curvature (in the Chern-basis [8]). Roughly speaking, the Dirac cones behave like $k$-space vortices that see the Chern-basis Berry curvature as a $k$-space magnetic field. The energetic competition between repulsion of vortices and a vortex tending to overlap magnetic fields, then determines the final location of the FB Dirac cones [9,11,20]. The uniformity of the Berry curvature in the FBs is mostly governed by the gap at the $\Gamma$ point to the dispersive bands. At zero gap, the Berry curvature is a delta function and the FB Dirac cones squeeze close to the $\Gamma$ point with larger FB $v_F$, whereas a gap increased the Berry curvature spread and the FB Dirac cones reside further from the $\Gamma$ point with lower FB $v_F$. Therefore the $\Gamma$ point gap size determines the low density FB Dirac cone $v_F$, which is what governs $dn_D/dn$, the quantity compared to experiment (Fig. 4f,g).

## V. Lack of gap at CNP

Experiment shows a significant density in the FB sector below the first peak in magnetization due to equilibrium currents flowing in the incompressible gap between the $N_D = 0$ and 1 LLs. At 56 mT (Fig. 4b), this can be approximated to be at an energy equal to $\varepsilon_1^D/2 = 3.9$ meV, implying a significant FB density below such a small energy.

As discussed in the main text, gaped states should generically be of order of the Coulomb scale, ~20 meV. This, coupled with the fact that the $D$ field dependance of the local LLs (Figs. 4a,b) give a $K$-point gap of the theoretically expected order (±19 meV), is strong evidence of the lack of a gap at CNP in our device.

Additionally, other local probes, such as STM have measured the gap at $\nu = 2$ in MATBG of order 10 meV [21], which is likely an underestimate due to the metallic screening of the STM tip. This is again in agreement with the theoretical order. Although some transport measurements at $\nu = 2$ measure smaller gaps (~few meV), this should be considered as a lower bound due to averaging over µm scale in systems that have large disorder, which a local probe avoids.

To put a more stringent bound on a possible gap in the system, we preform simulations of the QOs in magnetization on a simplified model and compare to experimental $B_z^{ac}(n)$ (Extended Data Fig. 6). To model the FB at low densities we take two Dirac cones with renormalized $v_F^{FB} \approx v_F/3.5$, and calculate the QOs arising from the LLs in the Dirac sector broadened by the Dingle $\Gamma \cong 1$ meV, as described in [22]. To best match the experiment we add some small mass to the FB cones, such that the zero crossing is not linear. This implies an enhanced DOS in the FB at CNP that can be explained either by disorder or by a NSM state in which the FB Dirac cones move very close to each other such that they hybridize and effectively have a parabolic dispersion at very low energy. Finally, we allow a gap $\Delta$ to open in the FB and extract $\partial M/\partial n \propto B_z^{ac}$ by convolving the calculated magnetization with our $ac$ density excitation, $n^{ac}$. With increasing $\Delta$, a sharp peak appears at $n \approx 0.04 \times 10^{12}$ (Extended Data Fig. 6b), which is not present in experiment. Even at $\Delta = 1$ meV (red), a small peak is discerned in simulations. Therefore, we place as a conservative estimate, $\Delta < 1$ meV. Note that the steep initial rise of the calculated $\partial M/\partial n$ cresting at the peak, in contrast to the much more gradual initial increase in the experimental $B_z^{ac}$, is the result of rapid initial filling of the Dirac band for finite $\Delta$ due to the absence of DOS in the FB.

A very small gap of 1 meV or smaller cannot be completely ruled out experimentally. Indeed, there can be a second order phase transition between the NSM and KIVC state, tuned by the strength of the strain [19]. In this scenario, the state would have both a NSM order parameter (which is $C_3$ breaking) and a KIVC order parameter, where the NSM order parameter is much larger than the KIVC. Effectively this can be thought of as an NSM that breaks $C_3$ with a small mass term induced by the small KIVC order parameter. Therefore, even if there were to be a small FB gap that experiment is not sensitive to, the state should still be thought of as a NSM from a theoretical perspective.

## VI. Fitting BS parameters

The $B_a = 251$ mT experimental data and derived $dn_D/dn$ in Fig. 2 allow for sensitive fitting of BS parameters [22,23] with the information from the FBs and up through the dispersive bands ($\nu > 4.2$). Particularly, there are three features that we would like to fit:

1. The maximal value of $dn_D/dn \approx 0.1$ at the top of the FB.
2. The lack of a large gap to the dispersive band in the FB sector. A large gap would result in $dn_D/dn = 1$ in the gap, since there would be only the graphene Dirac cone Fermi surface, which is not observed.
3. Increased $dn_D/dn \approx 0.2$ in the dispersive bands.

Additionally, we have the $B_a = 56$ mT experimental data and derived $dn_D/dn$ in Fig. 4f with information about the physics near CNP. As described in the previous section, the gap to the dispersive band at $\nu = 0$ is related to $v_F$ of the FB Dirac cones in the NSM state, and therefore to the DOS of the FBs near CNP. We

find that with no gap, $dn_D/dn$ is too large (~0.1). Therefore, to fit both high and low field data (or equivalently, both high and low energy physics), we need a clear single particle gap to the dispersive bands at $\nu = 0$, that is not too large when reaching $\nu \approx 4$ from the added Hartree potential (Figs. 2h-j).

We assign the generally accepted value $\gamma_0 = 2800$ as a starting point and span the generally expected range of the other parameters. We find that using $w_1$ = 110 meV, $w_0 = 0.72w_1$, and $\epsilon \approx 17$ matches both the high and low energy physics. Due to the large number of parameters compared to the number of features, there can be other parameter sets that would match qualitatively our data.

Note that it would not be surprising if parameters that yield zero gap at $\nu \approx 4$ give only a qualitatively accurate DOS at charge neutrality. There are many effects in practice that the BM model does not take into account, such as $C_3$ symmetric strain due to lattice relaxation, as well as heterostrain. Additionally, there is no source of particle-hole symmetry breaking. These could have distinct quantitative effects, that are hard to predict on these two very different states – NSM at charge neutrality and the Hartree renormalized BM bands at $\nu \approx 4$. Furthermore, the sparse $k$-space grid for HF simulations leads to inherent numerical uncertainty and should only be taken qualitatively. For these reasons, qualitative agreement is the realistic measure in FB graphene systems. We are not aware of any works that have good quantitative agreement across such different filling factors for the same parameters.

**Supplementary information references**


1. Y. Cao, V. Fatemi, S. Fang, K. Watanabe, T. Taniguchi, E. Kaxiras, and P. Jarillo-Herrero, ''Unconventional superconductivity in magic-angle graphene superlattices'', *Nature* **556**, 43 (2018).
2. J. M. Park, Y. Cao, K. Watanabe, T. Taniguchi, and P. Jarillo-Herrero, ''Tunable strongly coupled superconductivity in magic-angle twisted trilayer graphene'', *Nature* **590**, 249 (2021).
3. A. T. Pierce, Y. Xie, J. M. Park, E. Khalaf, S. H. Lee, Y. Cao, D. E. Parker, P. R. Forrester, S. Chen, K. Watanabe, T. Taniguchi, A. Vishwanath, P. Jarillo-Herrero, and A. Yacoby, ''Unconventional sequence of correlated Chern insulators in magic-angle twisted bilayer graphene'', *Nat. Phys.* **17**, 1210 (2021).
4. R. Bistritzer and A. H. MacDonald, ''Moiré bands in twisted double-layer graphene'', *Proc. Natl. Acad. Sci.* **108**, 12233 (2011).
5. E. Khalaf, A. J. Kruchkov, G. Tarnopolsky, and A. Vishwanath, ''Magic angle hierarchy in twisted graphene multilayers'', *Phys. Rev. B* **100**, 85109 (2019).
6. F. Guinea and N. R. Walet, ''Electrostatic effects, band distortions, and superconductivity in twisted graphene bilayers'', *Proc. Natl. Acad. Sci. U. S. A.* **115**, 13174 (2018).
7. M. Xie and A. H. Macdonald, ''Nature of the Correlated Insulator States in Twisted Bilayer Graphene'', *Phys. Rev. Lett.* **124**, 97601 (2020).
8. N. Bultinck, E. Khalaf, S. Liu, S. Chatterjee, A. Vishwanath, and M. P. Zaletel, ''Ground State and Hidden Symmetry of Magic-Angle Graphene at Even Integer Filling'', *Phys. Rev. X* **10**, 031034 (2020).
9. S. Liu, E. Khalaf, J. Y. Lee, and A. Vishwanath, ''Nematic topological semimetal and insulator in magic-angle bilayer graphene at charge neutrality'', *Phys. Rev. Res.* **3**, 1 (2021).
10. T. Cea and F. Guinea, ''Band structure and insulating states driven by Coulomb interaction in twisted bilayer graphene'', *Phys. Rev. B* **102**, 2 (2020).
11. P. J. Ledwith, E. Khalaf, Z. Zhu, S. Carr, E. Kaxiras, and A. Vishwanath, ''TB or not TB? Contrasting properties of twisted bilayer graphene and the alternating twist n-layer structures (n=3, 4, 5, ...)'', *arXiv:2111.11060* (2021).
12. J. S. Hofmann, E. Khalaf, A. Vishwanath, E. Berg, and J. Y. Lee, ''Fermionic Monte Carlo Study of a Realistic Model of Twisted Bilayer Graphene'', *Phys. Rev. X* **12**, 11061 (2022).
13. D. Parker, P. Ledwith, E. Khalaf, T. Soejima, J. Hauschild, Y. Xie, A. Pierce, M. P. Zaletel, A. Yacoby, and A. Vishwanath, ''Field-tuned and zero-field fractional Chern insulators in magic angle

graphene'', *arxiv:2112.13837* (2021).
14. F. Xie, N. Regnault, D. Călugăru, B. A. Bernevig, and B. Lian, ''Twisted symmetric trilayer graphene. II. Projected Hartree-Fock study'', *Phys. Rev. B* **104**, 1 (2021).
15. M. Christos, S. Sachdev, and M. S. Scheurer, ''Correlated Insulators, Semimetals, and Superconductivity in Twisted Trilayer Graphene'', *Phys. Rev. X* **12**, 21018 (2022).
16. B. Lian, Z. Da Song, N. Regnault, D. K. Efetov, A. Yazdani, and B. A. Bernevig, ''Twisted bilayer graphene. IV. Exact insulator ground states and phase diagram'', *Phys. Rev. B* **103**, 1 (2021).
17. P. J. Ledwith, E. Khalaf, and A. Vishwanath, ''Strong coupling theory of magic-angle graphene: A pedagogical introduction'', *Ann. Phys. (N. Y).* **435**, 168646 (2021).
18. O. Vafek and J. Kang, ''Renormalization Group Study of Hidden Symmetry in Twisted Bilayer Graphene with Coulomb Interactions'', *Phys. Rev. Lett.* **125**, 257602 (2020).
19. D. E. Parker, T. Soejima, J. Hauschild, M. P. Zaletel, and N. Bultinck, ''Strain-Induced Quantum Phase Transitions in Magic-Angle Graphene'', *Phys. Rev. Lett.* **127**, 27601 (2021).
20. E. Khalaf, N. Bultinck, A. Vishwanath, and M. P. Zaletel, ''Soft modes in magic angle twisted bilayer graphene'', *arXiv:2009.14827v2* (2020).
21. K. P. Nuckolls, R. L. Lee, M. Oh, D. Wong, T. Soejima, J. P. Hong, D. Călugăru, J. Herzog-Arbeitman, B. A. Bernevig, K. Watanabe, T. Taniguchi, N. Regnault, M. P. Zaletel, and A. Yazdani, ''Quantum textures of the many-body wavefunctions in magic-angle graphene'', *Nature* **620**, 525 (2023).
22. H. Zhou, N. Auerbach, M. Uzan, Y. Zhou, N. Banu, W. Zhi, M. E. Huber, K. Watanabe, T. Taniguchi, Y. Myasoedov, B. Yan, and E. Zeldov, ''Imaging quantum oscillations and millitesla pseudomagnetic fields in graphene'', *Nature* **624**, 275 (2023).
23. M. Bocarsly, M. Uzan, I. Roy, S. Grover, J. Xiao, Z. Dong, M. Labendik, A. Ur, M. E. Huber, Y. Myasoedov, K. Watanabe, T. Taniguchi, B. Yan, L. S. Levitov, and E. Zeldov, ''De Haas – van Alphen spectroscopy and magnetic breakdown in moiré graphene'', *Science* **383**, 42 (2024).
